# Association between C-reactive protein-triglyceride glucose index and all-cause mortality and premature death: a joint analysis based on case data from the Central Hospital of Shaoyang and CHARLS database

**DOI:** 10.3389/fmed.2025.1656187

**Published:** 2025-10-28

**Authors:** Tao Sun, Manke Zhang, Jun Liu, Zhen An

**Affiliations:** ^1^Department of Hematology and Oncology Laboratory, The Central Hospital of Shaoyang, Shaoyang, Hunan, China; ^2^Department of Nursing, Chenzhou Third People’s Hospital, Chenzhou, Hunan, China; ^3^Department of Scientific Research, The First Affiliated Hospital of Shaoyang University, Shaoyang, Hunan, China

**Keywords:** C-reactive protein-triglyceride glucose index, all-cause mortality, premature death, multicenter study, CHARLS, clinical cohort, Cox regression, restricted cubic spline analysis

## Abstract

**Background:**

This study systematically investigates the relationship between C-reactive protein-triglyceride glucose index (CTI) and the risks of all-cause and premature mortality.

**Methods:**

A total of 10,350 participants from the China Health and Retirement Longitudinal Study (CHARLS) (2011–2020) and 1,842 participants from the Central Hospital of Shaoyang (CHSY) (2019–2024), aged 45 years or older, were included. CTI was calculated based on C-reactive protein (CRP) and the triglyceride-glucose index (TyG). Cox proportional hazard models were employed to assess the association between CTI and all-cause mortality and premature death. Restricted cubic spline (RCS) analysis were used to explore potential non-linear relationships. Subgroup and sensitivity analyses were conducted to verify the robustness of the findings. In addition, the concordance index (C-index) evaluate the risk differentiation ability of the different indicators.

**Results:**

In the CHARLS cohort, each one-standard-deviation increase in the CTI was associated with an elevated risk of mortality (all-cause mortality: HR = 1.86; premature death: HR = 2.10). Similar results were observed in the CHSY cohort (all-cause mortality: HR = 1.84; premature death: HR = 2.37). Restricted cubic spline analysis revealed a non-linear dose–response relationship in the CHSY dataset. Subgroup analyses indicated that this association was more pronounced among males, individuals with lower education levels, and those without hypertension. Sensitivity analyses yielded consistent results, supporting the robustness of the findings. In terms of predictive performance, C-index analysis demonstrated that the discriminative ability of CTI was slightly superior to that of the TyG index (mostly ranging between 0.61 and 0.65), suggesting its potential utility in risk prediction.

**Conclusion:**

This multicenter pooled analysis provides evidence that elevated CTI is an independent risk factor for all-cause and premature mortality, supporting its potential utility in public health screening and clinical risk assessment. However, further prospective studies are warranted to validate its clinical applicability.

## 1 Introduction

The synergistic interplay between metabolic disorders and chronic inflammation represents a fundamental driver of the global disease burden (1, 2). Data from the World Health Organization (WHO) indicate that diseases related to metabolic dysfunction, such as diabetes and cardiovascular diseases, are responsible for over 19 million deaths annually. This accounts for more than 35% of all-cause mortality, with notably higher risks of premature mortality observed in low- and middle-income countries (3, 4). Although traditional biomarkers, such as fasting glucose (GLU) and triglycerides (TG), are commonly employed in risk assessment, their limited sensitivity has become increasingly apparent, particularly in identifying high-risk populations experiencing metabolic-inflammatory interactions (5, 6). In recent years, the development of composite biomarkers has emerged as a pivotal focus in precision medicine, as the integration of metabolic and inflammatory indicators facilitates a more comprehensive evaluation of health risks (7).

C-reactive protein-triglyceride glucose index (CTI) represents an innovative composite marker that integrates key components - C-reactive protein (CRP), triglycerides, and glucose—each of which has been robustly associated with diabetes, non-alcoholic fatty liver disease, and cardiovascular events (8–10). Nevertheless, current research predominantly focuses on single cohort studies, lacking comprehensive validation across both clinical and community populations (11). Furthermore, the prognostic utility of the CTI in predicting all-cause and premature mortality necessitates further corroboration through multi-source data. Existing evidence indicates substantial limitations in mortality risk prediction when relying exclusively on individual metabolic or inflammatory markers. In recent years, the triglyceride-glucose (TyG) index has gained increasing attention for its value in disease prediction, demonstrating significant potential as a screening biomarker for various conditions, including chronic kidney disease, type 2 diabetes, ischemic stroke, and non-alcoholic fatty liver disease (12). Although the TyG index effectively reflects insulin resistance, it does not incorporate inflammatory markers, which may lead to an underestimation of risk in patients with coexisting chronic inflammation (13). On the other hand, CRP, a traditional inflammatory marker, demonstrates predictive capabilities that are potentially confounded by variables such as obesity and metabolic status (14). By integrating TyG and CRP, the CTI is theoretically designed to capture the synergistic effects of metabolic dysregulation and low-grade inflammation (15). However, the majority of existing studies predominantly focus on cross-sectional associations between CTI and specific diseases, with data primarily derived from public databases and lacking longitudinal evidence on cross-population consistency (16). Furthermore, many analyses presuppose a linear relationship between CTI and outcomes, potentially neglecting threshold effects or U-shaped associations, which may compromise the accuracy of risk stratification (17). Significantly, determinants of mortality risk may vary across subgroups with different levels of education and underlying comorbidities, yet evidence regarding CTI subgroup analyses remains limited.

The complementary characteristics of the CHARLS and hospital-based cohort data provide a valuable opportunity to address existing limitations. CHARLS, as a nationally representative and large-scale cohort, offers extensive data on metabolic indicators, inflammatory markers, and long-term mortality outcomes (18). In contrast, the CHSY cohort delivers high-precision metabolic-inflammatory measurements within clinical environments. Although previous studies have employed CHARLS to investigate the associations between metabolic markers and health outcomes (19–21), no research has yet integrated hospital data to assess the generalizability of the predictive efficacy of CTI. Furthermore, there has been no systematic comparison of the differences between clinical and community populations. This knowledge gap impedes the application of CTI in public health practice, particularly in the development of low-cost screening tools for resource-constrained regions.

This study combines case data from the Central Hospital of Shaoyang with CHARLS to address the following key questions: (1) Whether CTI independently predicts all-cause and premature mortality risks in both clinical and community populations; (2) Whether the association between CTI and mortality exhibits cohort-specific non-linear patterns; and (3) Differences in CTI’s predictive performance across healthcare settings (hospital vs. community) and population subgroups. Through the synthesis of multicenter data, Cox proportional hazards models, and restricted cubic spline analyses, this study assesses the cross-cohort predictive value of CTI in the Chinese population. The findings will provide stratified evidence for the clinical utility of metabolic-inflammatory composite markers and inform the development of tailored screening strategies.

## 2 Materials and Methods

### 2.1 Study design

**FIGURE 1:**
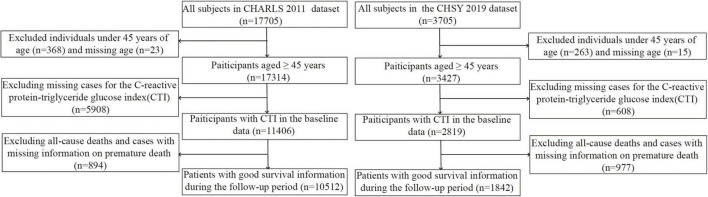
Flow chart of the selection of study participants.

This research utilized data from the CHARLS covering the period from 2011 to 2020, as well as the CHSY cohort from 2019 to 2024. CHARLS is a nationally representative longitudinal cohort study that focuses on middle-aged and elderly populations aged 45 years and above across 28 provinces in China. It employs a multistage stratified sampling method to ensure the sample’s representativeness (18). The baseline survey of CHARLS, conducted in 2011, initially included 17,705 participants. Following data cleaning, 10,512 eligible subjects were retained. The exclusion criteria were as follows: (1) absence of key variables, such as CTI-related indicators and mortality data, (2) missing age information or age below 45 years, and (3) missing covariates exceeding 30%. These criteria were similarly applied to the hospital cohort. The CHSY cohort initially enrolled 3,705 hospitalized individuals in 2019, with 1,858 subjects retained after screening. Details regarding missing data rates are provided in Supplementary Figure 1. To address potential selection bias arising from missing data, we generated five imputed datasets using Multiple Imputation by Chained Equations (MICE). All analyses were performed separately within each imputed dataset, and the results were then synthesized using Rubin’s rules to obtain pooled estimates and standard errors (22). During the data preprocessing phase, several analytical procedures were undertaken, including outlier handling, multicollinearity analysis, variable correlation assessment, and normality testing. Multicollinearity was assessed using the variance inflation factor (VIF), and variables exhibiting a VIF greater than 10 were excluded from further analysis. To evaluate intervariable associations, Spearman or Pearson correlation analysis were conducted. The normality of the data was assessed using the Kolmogorov-Smirnov test to ensure compliance with statistical assumptions. Both cohorts underwent unit standardization, with CRP, TG, and GLU uniformly converted to milligrams per deciliter (mg/dL). The flowchart detailing participant selection is presented in Figure 1.

### 2.2 Study variables

#### 2.2.1 Exposure factor

The primary exposure variable was the CTI, which was constructed based on the following three dimensions: First, the TyG was calculated using fasting TG and GLU levels, with the formula as follows (23):


$$T\,y\,G=\ln\,\left(\frac{\mathrm{TG}\,\left(\mathrm{mg}/\mathrm{dL}\right)\times \mathrm{Glucose}\,\left(\mathrm{mg}/\mathrm{dL}\right)}{2}\right)$$

C-reactive protein levels in the CHARLS cohort were determined using latex-enhanced immunonephelometry, with a detection sensitivity of 0.1 mg/L and an intra-assay coefficient of variation (CV) of less than 5% (3). In the CHSY cohort, CRP levels were measured via immunoturbidimetry (Roche Cobas c702, CV < 3%). The CTI formula, including the coefficient 0.412 for log (CRP), was adopted from Ruan et al. (24), where it was derived and validated for mortality prediction in cancer populations. The specific formula for the CTI index was as follows:


C⁢T⁢I=0.412×log⁢(CRP⁢(mg/L))+T⁢y⁢G

#### 2.2.2 Outcome events

The primary outcome assessed was all-cause mortality within the CHARLS cohort and the CHSY cohort. The secondary outcome focused on premature mortality within each cohort. Information on mortality in the CHARLS cohort was obtained from follow-up records during the first follow-up (2013, Wave 1, approximately 2 years after the 2011 baseline) and the second follow-up (2020, Wave 2, approximately 9 years after the 2011 baseline). Death status was reported by family members and verified through the household registration system. For the CHSY cohort, mortality data were confirmed through the hospital information system. Premature mortality was defined as all-cause mortality occurring before the age of 72.7 years for males and 76.9 years for females, based on China’s average life expectancy in 2011 (25, 26). The follow-up duration for deceased individuals was calculated as the year of death minus the baseline year (2011 for CHARLS and 2019 for CHSY). The follow-up period spanned 10 years for participants in the CHARLS cohort and 5 years for those in the CHSY cohort.

#### 2.2.3 Covariates

To address potential confounding effects, the analysis incorporated the following covariates: demographic characteristics (age, sex, education level, marital status, household registration), lifestyle factors (smoking, alcohol consumption), medical history (diabetes (DM), hypertension (HTN), coronary heart disease (CVD)), clinical indicators (body mass index (BMI)), and laboratory parameters (TG, high-density lipoprotein (HDL-C), low-density lipoprotein (LDL-C), GLU, urine acid (UA)). Comprehensive definitions, attributes, and value assignments for these covariates are detailed in Supplementary Table 1.

### 2.3 Statistical analysis

Missing values were addressed through the application of MICE (27), while outliers were detected using the winsorization method (28), each continuous variable was truncated at the 1% and 99% quartiles. Multicollinearity among the independent variables was evaluated using the VIF (29). The normality of continuous variables was assessed using the Kolmogorov-Smirnov test (using Lilliefors correction for normality) (30). Continuous variables were reported as mean ± standard deviation or median (interquartile range), with between-group comparisons conducted using the Wilcoxon rank-sum test or the Kruskal-Wallis test. Categorical variables were presented as frequencies (percentages), with between-group comparisons performed using Fisher’s exact test when expected frequencies were less than 5; otherwise, the chi-square test was utilized. A two-stage analytical strategy was implemented. To verify the prerequisite conditions of the Cox regression model, we conducted proportional hazards (PH) assumption tests for all fitted models. Specifically, global and covariate-specific tests were performed using Schoenfeld residual tests (via the cox.zph() function), supplemented by visual inspection of residual plots to assess whether variable effects remained stable over the follow-up period. The Cox proportional hazards models adjusted for confounding variables in three stages. In Model 1, no covariates were included. Model 2 incorporated adjustments for age, sex, BMI, education level, marital status, household registration (hukou), smoking habits, alcohol consumption, hypertension, diabetes, and cardiovascular disease. Model 3 included further adjustments for TG, LDL, HDL, UA, and GLU. To explore potential non-linear relationships between CTI and mortality risk, RCS were employed with varying numbers of knots (3, 4, 5, 6, 7) (31). In the CHARLS cohort, the number of knots was tested from 3 to 7, and the optimal number for each outcome was selected based on the minimum Akaike Information Criterion (AIC): 4 knots were used for all-cause mortality in 2013, 3 knots for premature mortality in 2013, 4 knots for all-cause mortality in 2020, and 3 knots for premature mortality in 2020. Knot locations were automatically assigned by the rms package based on percentiles of the predictor distribution. In the CHSY cohort, knots were pre-specified at the 5th, 35th, 65th, and 95th percentiles of the predictor distribution to ensure comparability across analyses (32). Subgroup analyses were conducted, stratified by sex, education level, marital status, household registration, smoking history, alcohol consumption, and baseline disease status, with interaction terms employed to assess effect heterogeneity. We conducted sensitivity analyses using three distinct strategies: (1) a dataset excluding participants with chronic diseases such as hypertension, diabetes, and coronary heart disease; (2) a dataset derived from a complete-case analysis, which involved the direct deletion of missing values rather than the application of multiple imputation techniques; and (3) the removal of certain metabolic indicators (e.g., TG, LDL-C, HDL-C, GLU) that may lie on the causal pathway during model construction to avoid potential overadjustment effects, thereby examining the robustness of the association between CTI and mortality risk. To evaluate the discriminative ability of different indicators for the outcome events (all-cause death and premature death), this study calculated the concordance index (C-index) using the survival package. All statistical analyses were executed using R software (version 4.2.2). Cox proportional hazards models were implemented through the “survival” package (version 3.6.4), while RCS analysis was conducted using the “rms” package (version 6.8.2). Additionally, multicollinearity was assessed with the aid of the “car” (version 3.1.3) and “boot” (version 1.3.30) packages. A two-sided *p*-value of less than 0.05 was deemed to indicate statistical significance.

## 3 Results

### 3.1 Baseline characteristics analysis

To further investigate the interrelationships among variables, this study conducted correlation analysis, multicollinearity testing, and normality testing based on the CHARLS cohort. Multicollinearity analysis (Supplementary Table 2) revealed that the variance inflation factor (VIF) for all variables was below 5, ranging from 1.06 to 3.24, which is within an acceptable range. CTI and TG exhibited the highest VIF values (3.24 and 2.53, respectively), while demographic variables (such as age, sex, and education level) all had VIF values below 2.5, indicating no severe multicollinearity in the model. Correlation analysis (Supplementary Figure 2) showed that CTI was strongly positively correlated with TG (*r* = 0.74) and GLU (*r* = 0.52), and strongly negatively correlated with HDL (*r* = −0.49); it was moderately positively correlated with hypertension, diabetes, and UA (*r* = 0.26–0.47). Normality testing (Supplementary Table 3) indicated that none of the continuous variables followed a normal distribution.

Baseline characteristics stratified by CTI quartiles (Table 1) revealed significant differences across CTI groups in demographic, clinical, and lifestyle factors. With increasing CTI, age, BMI, and lipid profiles (TG, LDL) showed a progressive upward trend (all *p* < 0.001). The proportion of females increased from 49.90% in Q1 to 55.49% in Q4, while the proportions of males, drinkers, and smokers gradually decreased. The prevalence of hypertension (57.37%), diabetes (19.54%), and cardiovascular disease (19.01%) in Q4 was significantly higher than that in Q1 (30.56%, 3.22%, and 11.34%, respectively). No significant differences were observed in education level or marital status. Both all-cause mortality and premature mortality in 2013 and 2020 increased with higher CTI levels, with Q4 exhibiting the highest rates (all *p* < 0.001). Baseline analyses based on survival outcomes in 2013 and 2020 (Supplementary Tables 4, 5) further indicated that individuals with high CTI had significantly higher all-cause mortality and premature mortality, with more pronounced differences in 2020 compared to 2013 (*p* < 0.01). In the CHSY database (Supplementary Table 6), a total of 1,842 patients were included. After stratification by CTI quartiles, the distributions of age (approximately 65–66 years), sex (male approximately 54%–56%), BMI, smoking and alcohol consumption status, as well as the prevalence of hypertension and diabetes, were generally balanced across the groups. Some differences were observed in urban versus rural household registration and history of cardiovascular disease among the groups, although the overall differences were minor. Regarding biochemical indicators, TG and GLU significantly increased with higher CTI, while HDL levels progressively decreased, both LDL and UA exhibited mild elevations.

**TABLE 1:** Patient demographics and baseline characteristics in the CHARLS dataset.

Variables	CTI				*p*-value
	Q1, *N* = 2,611^1^	Q2, *N* = 2,620^1^	Q3, *N* = 2,630^1^	Q4, *N* = 2,651^1^	
Age	57.00 (50.00, 64.00)	59.00 (52.00, 66.00)	59.00 (53.00, 66.00)	59.00 (53.00, 66.00)	<0.001^1^
Gender					<0.001^2^
Female	1,303 (49.90%)	1,355 (51.72%)	1,447 (55.02%)	1,471 (55.49%)	
Male	1,308 (50.10%)	1,265 (48.28%)	1,183 (44.98%)	1,180 (44.51%)	
BMI	21.86 (19.96, 23.96)	22.61 (20.51, 25.03)	23.88 (21.51, 26.45)	24.91 (22.47, 27.72)	<0.001^1^
Education					0.265^2^
College/Uni+	41 (1.57%)	33 (1.26%)	41 (1.56%)	47 (1.77%)	
Illiterate	744 (28.49%)	787 (30.04%)	776 (29.51%)	763 (28.78%)	
Primary	1,048 (40.14%)	1,098 (41.91%)	1,065 (40.49%)	1,050 (39.61%)	
Second/high school	778 (29.80%)	702 (26.79%)	748 (28.44%)	791 (29.84%)	
Marital					0.069^2^
Divorced	32 (1.23%)	26 (0.99%)	30 (1.14%)	22 (0.83%)	
Married	2,324 (89.01%)	2,312 (88.24%)	2,284 (86.84%)	2,324 (87.67%)	
Unmarried	27 (1.03%)	22 (0.84%)	16 (0.61%)	23 (0.87%)	
Widowed	228 (8.73%)	260 (9.92%)	300 (11.41%)	282 (10.64%)	
Hukou					<0.001^2^ <0.001^2^
Town	400 (15.32%)	402 (15.34%)	491 (18.67%)	620 (23.39%)	
Village	2,211 (84.68%)	2,218 (84.66%)	2,139 (81.33%)	2,031 (76.61%)	
Smoking					
Ex-smoker	197 (7.55%)	236 (9.01%)	241 (9.16%)	266 (10.03%)	
Non-smoker	1,572 (60.21%)	1,577 (60.19%)	1,628 (61.90%)	1,662 (62.69%)	
Smoker	842 (32.25%)	807 (30.80%)	761 (28.94%)	723 (27.27%)	
Drinking					<0.001^2^
No	1,659 (63.54%)	1,732 (66.11%)	1,825 (69.39%)	1,842 (69.48%)	
Yes	952 (36.46%)	888 (33.89%)	805 (30.61%)	809 (30.52%)	
HTN					<0.001^2^
No	1,813 (69.44%)	1,600 (61.07%)	1,384 (52.62%)	1,130 (42.63%)	
Yes	798 (30.56%)	1,020 (38.93%)	1,246 (47.38%)	1,521 (57.37%)	
DM					<0.001^2^
No	2,527 (96.78%)	2,513 (95.92%)	2,433 (92.51%)	2,133 (80.46%)	
Yes	84 (3.22%)	107 (4.08%)	197 (7.49%)	518 (19.54%)	
CVD					<0.001^2^
No	2,315 (88.66%)	2,296 (87.63%)	2,233 (84.90%)	2,147 (80.99%)	
Yes	296 (11.34%)	324 (12.37%)	397 (15.10%)	504 (19.01%)	
TG	67.26 (54.87, 81.42)	95.58 (77.00, 115.94)	128.33 (100.01, 159.30)	199.13 (141.60, 278.78)	<0.001^1^
LDL	108.64 (90.46, 127.97)	116.37 (95.88, 137.63)	119.07 (97.81, 141.50)	113.27 (86.99, 141.11)	<0.001^1^
HDL	58.38 (49.49, 68.43)	52.19 (44.85, 61.47)	46.78 (39.43, 55.67)	39.43 (32.86, 47.94)	<0.001^1^
UA	3.99 (3.34, 4.76)	4.21 (3.54, 5.07)	4.39 (3.67, 5.24)	4.67 (3.85, 5.59)	<0.001^1^
GLU	95.94 (89.10, 103.50)	100.26 (93.60, 107.82)	103.68 (96.12, 113.76)	114.84 (103.14, 141.12)	<0.001^1^
All cause mortality_2013					<0.001^2^
No	2,585 (99.00%)	2,592 (98.93%)	2,587 (98.37%)	2,574 (97.10%)	
Yes	26 (1.00%)	28 (1.07%)	43 (1.63%)	77 (2.90%)	
Premature death_2013					<0.001^2^
No	2,598 (99.50%)	2,608 (99.54%)	2,608 (99.16%)	2,606 (98.30%)	
Yes	13 (0.50%)	12 (0.46%)	22 (0.84%)	45 (1.70%)	
All cause mortality_2020					<0.001^2^
No	2,573 (98.54%)	2,577 (98.36%)	2,574 (97.87%)	2,559 (96.53%)	
Yes	38 (1.46%)	43 (1.64%)	56 (2.13%)	92 (3.47%)	
Premature death_2020					<0.001^2^
No	2,592 (99.27%)	2,602 (99.31%)	2,601 (98.90%)	2,602 (98.15%)	
Yes	19 (0.73%)	18 (0.69%)	29 (1.10%)	49 (1.85%)	

^1^Kruskal-Wallis rank sum test;

^2^Pearson’s Chi-squared test.

### 3.2 Association between CTI and all-cause mortality/premature mortality

The association between CTI and both all-cause and premature mortality was investigated using two distinct population cohorts: the CHARLS and CHSY, as detailed in Table 2. Within the CHARLS cohort, a significant positive correlation was identified between CTI and both all-cause and premature mortality. This relationship demonstrated a progressive strengthening across various adjusted models. Specifically, in the CHARLS data from the first follow-up (Wave 1, 2013, approximately 2 years of follow-up), the standardized CTI was associated with a HR of 2.15 (95% CI: 1.73–2.68, *p* < 0.001) for all-cause mortality and 2.44 (95% CI: 1.81–3.29, *p* < 0.001) for premature mortality in Model 3. Similarly, at the second follow-up (Wave 2, 2020, approximately 9 years of follow-up), the corresponding HRs were 1.86 (95% CI, 1.53–2.26) and 2.15 (95% CI, 1.64–2.82), respectively, with all associations achieving statistical significance (*p* < 0.001). A parallel trend was observed in the CHSY cohort, where CTI continued to exhibit a significant association with mortality outcomes. Specifically, each standard deviation increase in standardized CTI was associated with a 84% increased risk of all-cause mortality (HR = 1.84, 95% CI: 1.44–2.34, *p* < 0.001) and a 137% increased risk of premature mortality (HR = 2.37, 95% CI: 1.69–3.33, *p* < 0.001). These associations remained robust after adjustment for covariates across all three models. To enhance the comparability across different datasets, we report both the unit effect estimates and the standardized effect estimates of CTI (Supplementary Table 7). This table presents the hazard ratios for both the continuous and standardized CTI values, derived from all three models in the CHARLS 2013, CHARLS 2020, and CHSY datasets. To ensure the robustness of the Cox regression analysis, the proportional hazards (PH) assumption was evaluated using Schoenfeld residual tests. The global PH tests for all six models revealed no significant violations: all-cause mortality in CHARLS 2013 (*p* = 0.4005), premature mortality in CHARLS 2013 (*p* = 0.6421), all-cause mortality in CHARLS 2020 (*p* = 0.1903), premature mortality in CHARLS 2020 (*p* = 0.2186), all-cause mortality in CHSY (*p* = 0.1862), and premature mortality in CHSY (*p* = 0.2312). Furthermore, covariate-specific tests and residual plots confirmed time-constant hazard ratios, indicating that the proportional hazards assumption was satisfied for all models (Supplementary Figures 3–8).

**TABLE 2:** Association between CTI and survival.

Characteristic	Model 1		Model 2		Model 3	
	HR (95% CI)	*p*	HR (95% CI)	*p*	HR (95% CI)	*p*
**All cause mortality_2013 in CHARLS dataset**						
CTI (standardized)	1.52 (1.33–1.74)	<0.001	1.53 (1.31–1.79)	<0.001	2.15 (1.73–2.68)	<0.001
**Premature death_2013 in CHARLS dataset**						
CTI (standardized)	1.72 (1.43–2.06)	<0.001	1.70 (1.39–2.09)	<0.001	2.44 (1.81–3.29)	<0.001
**All cause mortality_2020 in CHARLS dataset**						
CTI (standardized)	1.40 (1.25–1.58)	<0.001	1.42 (1.24–1.63)	<0.001	1.86 (1.53–2.26)	<0.001
**Premature death_2020 in CHARLS dataset**						
CTI (standardized)	1.54 (1.30–1.82)	<0.001	1.59 (1.32–1.92)	<0.001	2.15 (1.64–2.82)	<0.001
**All cause mortality in CHSY dataset**						
CTI (standardized)	1.64 (1.31–2.05)	<0.001	1.79 (1.40–2.27)	<0.001	1.84 (1.44–2.34)	<0.001
**Premature death in CHSY dataset**						
CTI (standardized)	2.34 (1.68–3.27)	<0.001	2.40 (1.71–3.38)	<0.001	2.37 (1.69–3.33)	<0.001

Model 1: no covariates were adjusted, Model 2 in CHARLS dataset: adjusted for age, gender, BMI, education, marital, hukou, smoking, drinking, HTN, DM, and CVD, Model 3 in CHARLS dataset: adjusted for age, gender, BMI, education, marital, hukou, smoking, drinking, HTN, DM, CVD, TG, LDL, HDL, UA, and GLU. Model 2 in CHSY dataset: adjusted for age, gender, BMI, hukou, smoking, drinking, HTN, DM, and CVD, Model 3 in CHSY dataset: adjusted for age, gender, BMI, hukou, smoking, drinking, HTN, DM, CVD, TG, LDL, HDL, UA, and GLU.

### 3.3 Subgroup analysis

#### 3.3.1 Subgroup analysis of all-cause mortality association

**FIGURE 2:**
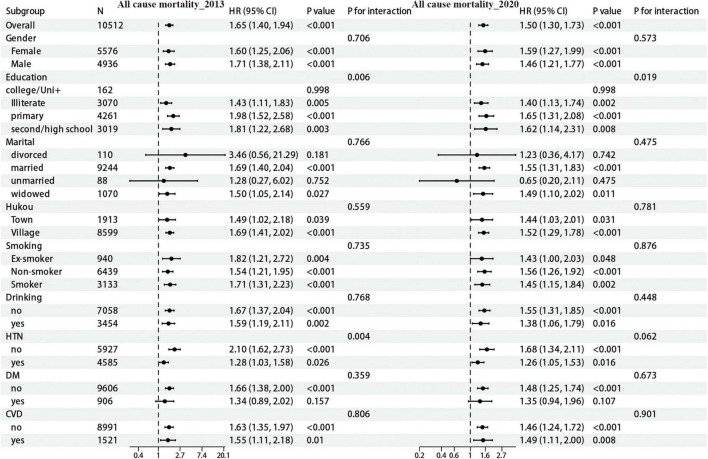
Subgroup analysis of CTI and risk of all-cause mortality (CHARLS cohort).

In the CHARLS cohort, follow-up data from 2013 indicated that an elevated CTI was significantly correlated with an increased risk of all-cause mortality (HR = 1.65, 95% CI: 1.40–1.94). This association demonstrated variability across subgroups stratified by demographic and clinical characteristics (Figure 2). A significant interaction was identified concerning educational attainment (*p* for interaction = 0.006), with the highest risk observed among individuals with only primary education (HR = 1.98, 95% CI: 1.52–2.58). Additionally, a significant interaction was observed for hypertension status (*p* for interaction = 0.004), revealing a higher risk among non-hypertensive individuals (HR = 2.10 vs. 1.28). An analysis of the 2020 follow-up data indicated a diminished association between the CTI and all-cause mortality (HR = 1.50, 95% CI: 1.30–1.73). The interaction effect of educational attainment demonstrated marginal significance (*p* for interaction = 0.019), with the primary education subgroup exhibiting the highest risk (HR = 1.65). Conversely, the interaction effect of hypertension status was no longer significant (*p* for interaction = 0.062). However, individuals without hypertension continued to show a higher risk (HR = 1.68 vs. 1.26). Within the CHSY cohort, a positive association between CTI and all-cause mortality was consistently observed across all subgroups (Supplementary Figure 9), with no statistically significant interactions identified. Stratified analyses revealed no significant interactions for sex (*p* for interaction = 0.993), hukou (*p* = 0.514), smoking status (*p* = 0.929), alcohol consumption (*p* = 0.835), hypertension (*p* = 0.572), diabetes (*p* = 0.560), or cardiovascular disease (*p* = 0.822), indicating a stable association between CTI and mortality risk within the CHSY population.

#### 3.3.2 Subgroup analysis of premature mortality association

**FIGURE 3:**
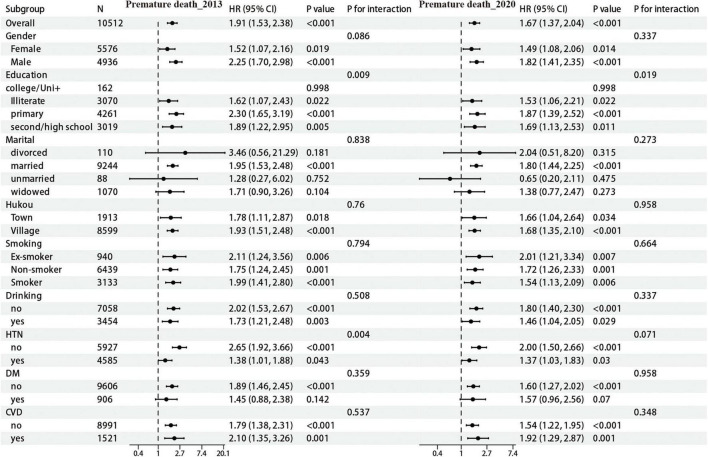
Subgroup analysis of CTI and risk of premature death (CHARLS cohort).

As illustrated in Figure 3, analysis of the follow-up data from the 2013 CHARLS cohort revealed a significant interaction effect for educational level (interaction *p*-value = 0.009), with the primary education subgroup exhibiting the highest risk (HR = 2.30). Furthermore, significant interactions was observed for hypertension (*p* for interaction = 0.004), with elevated risk noted among non-hypertensive individuals. Subsequent analysis of the 2020 data indicated that the association between the CTI and premature mortality remained significant (HR = 1.67, 95% CI: 1.37–2.04). The interaction effect related to educational attainment persisted (*p* = 0.019), with the primary education subgroup remaining the highest risk (HR = 1.87). Within the CHSY cohort, no statistically significant interactions were observed for sex (*p* for interaction = 0.728), hukou (*p* = 0.957), smoking (*p* = 0.286), alcohol consumption (*p* = 0.793), hypertension (*p* = 0.421), and cardiovascular disease (*p* = 0.619). Notably, a significant interaction was identified for diabetes (*p* for interaction = 0.042), with non-diabetic individuals exhibiting a higher risk of premature mortality (HR = 4.11).

### 3.4 Restricted cubic spline analysis

**FIGURE 4:**
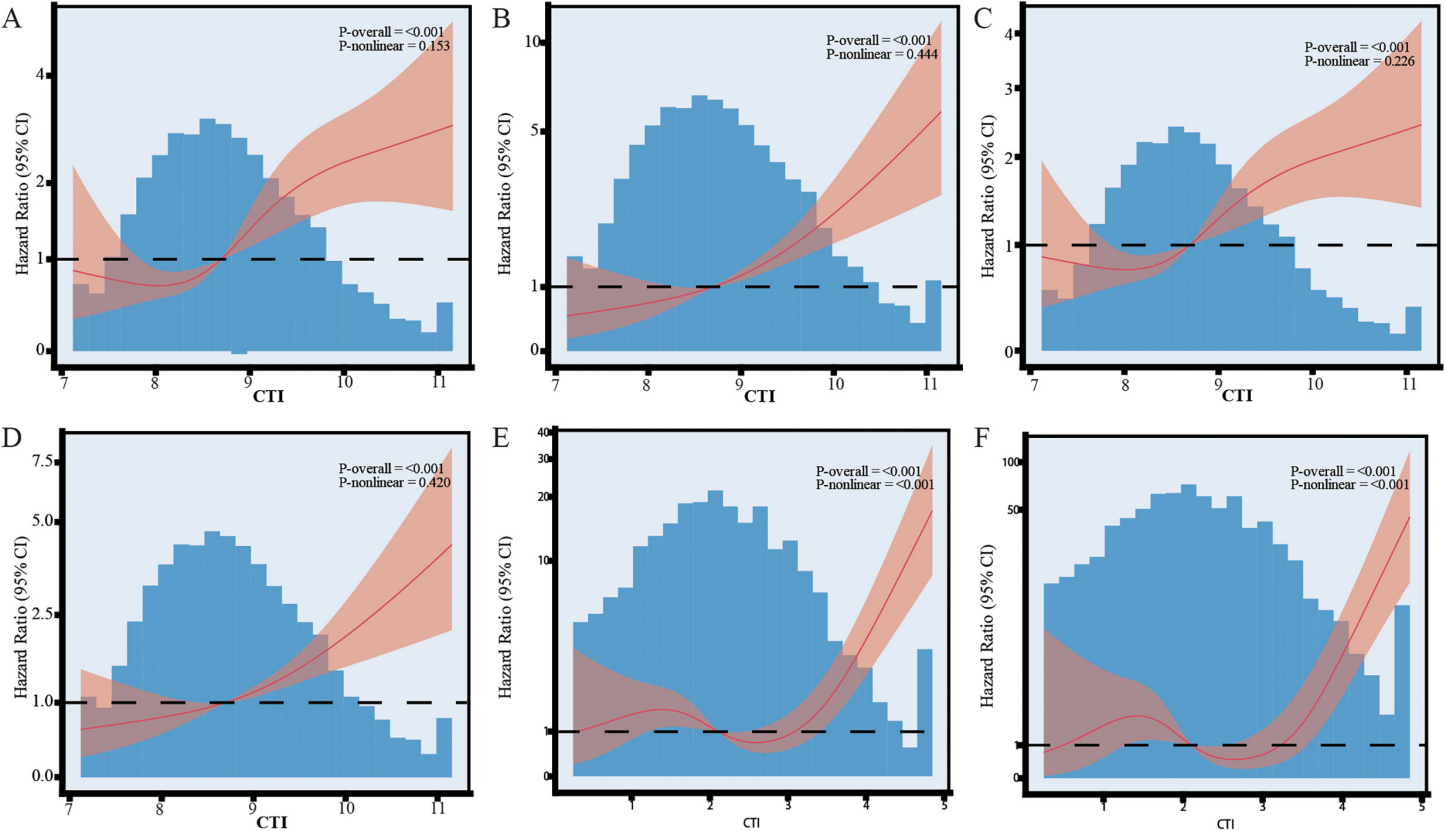
Non-linear associations between C-reactive protein-triglyceride glucose index (CTI) and the risks of all-cause mortality and premature mortality based on restricted cubic spline (RCS) models. **(A)** CTI and all-cause mortality in CHARLS 2013 follow-up; **(B)** CTI and premature mortality in CHARLS 2013 follow-up; **(C)** CTI and all-cause mortality in CHARLS 2020 follow-up; **(D)** CTI and premature mortality in CHARLS 2020 follow-up; **(E)** CTI and all-cause mortality in the CHSY cohort; **(F)** CTI and premature mortality in the CHSY cohort.

This study utilized the RCS analysis to investigate the dose-response relationship between the CTI and both all-cause and premature mortality. Models incorporating 3–7 knots were assessed, with the final number of knots determined by the lowest AIC value, as detailed in Supplementary Table 8. Figure 4 depicts the trends in the association between the CTI and the risk of endpoint events across different cohorts. In the CHARLS 2013 data (Figures 4A, B) and CHARLS 2020 data (Figures 4C, D), no significant non-linear relationship was observed (*P*-non-linear > 0.15), and mortality risk increased linearly with rising CTI levels. In the CHSY dataset (Figures 4E, F), CTI was also positively associated with all-cause and premature mortality risks; however, a significant non-linear effect was indicated (*P*-non-linear < 0.001), characterized by a relatively gradual increase in risk at lower CTI levels and a sharp rise at higher CTI levels. Overall, results from all three datasets consistently demonstrated that elevated CTI is closely associated with increased risks of all-cause and premature mortality, with a linear relationship observed in the CHARLS data and a potential non-linear dose–response pattern suggested in the CHSY data. The observed differences in thresholds compared to the CHARLS cohort may reflect variations in baseline metabolic-inflammatory status, health burden, and laboratory measurement conditions between clinical inpatients and community-dwelling populations. Therefore, caution should be exercised when applying CTI thresholds across different cohorts.

### 3.5 Sensitivity analysis

To ensure the robustness of the findings, three sensitivity analyses were performed. Firstly, an analysis utilizing complete-case data from the CHARLS, where missing values were excluded, indicated that the strength of the association between the CTI and each endpoint event was largely consistent with the primary analysis, as shown in Table 3. In 2013, the hazard ratios for standardized CTI about all-cause mortality across the three models were 1.48 (95% CI: 1.27–1.74), 1.45 (95% CI: 1.22–1.74), and 2.28 (95% CI: 1.76–2.96), all demonstrating statistically significant associations (*P* < 0.001). Additionally, a quartile-based analysis revealed a clear dose-response relationship (*P* for trend < 0.001), which persisted in the 2020 follow-up data, albeit with slightly reduced effect sizes. Secondly, in the analysis that excluded patients with hypertension, diabetes, or coronary heart disease (refer to Supplementary Table 9), the association between CTI and mortality risk was notably strengthened. In 2013, the standardized CTI hazard ratios for all-cause mortality increased to 1.71 (95% CI: 1.35–2.18), 1.98 (95% CI: 1.52–2.57), and 2.72 (95% CI: 1.91–3.87) across the three models. A comparable pattern of strengthened association was evident in the 2020 data, where the HRs for all-cause mortality and premature mortality in the model 3 were 2.21 and 2.34, respectively. These findings not only corroborate the robustness of the primary results but also reveal that CTI may have a more substantial predictive value for mortality risk in populations without pre-existing chronic diseases. Thirdly, to address potential overadjustment, a sensitivity analysis was conducted by excluding metabolic biomarkers (i.e., TG, LDL-C, HDL-C, and GLU) from the covariates in Model 3 (Supplementary Table 10). The results from both the CHARLS and CHSY datasets remained highly consistent with those of the primary analysis. For instance, in the CHARLS 2013 cohort, the HRs for all-cause mortality per unit increase in standardized CTI were 1.52 (95% CI: 1.33–1.74), 1.53 (95% CI: 1.31–1.79), and 1.56 (95% CI: 1.33–1.82) across the three models, all of which were statistically significant (*P* < 0.001). Similarly, during the CHARLS 2020 follow-up, the HRs for premature mortality ranged from 1.54 to 1.60 (all *P* < 0.001). A consistent pattern was also observed in the CHSY dataset, with HRs for both all-cause and premature mortality remaining stable (HRs ranging from 1.78 to 2.37, all *P* < 0.001). These findings suggest that the predictive ability of CTI is not entirely dependent on traditional metabolic markers, thereby reinforcing its robustness and independent prognostic value for adverse health outcomes.

**TABLE 3:** Association between CTI and survival in sensitivity analysis.

Characteristic	Model 1		Model 2		Model 3	
	HR (95% CI)^1^	*p*	HR (95% CI)^1^	*p*	HR (95% CI)^1^	*p*
**All cause mortality_2013**						
CTI (standardized)	1.48 (1.27–1.74)	<0.001	1.45 (1.22–1.74)	<0.001	2.28 (1.76–2.96)	<0.001
CTI						
Q1						
Q2	0.95 (0.51–1.77)	0.863	0.83 (0.44–1.56)	0.568	0.97 (1.07–2.60)	0.919
Q3	1.70 (0.98–2.96)	0.059	1.45 (0.83–2.54)	0.190	1.94 (1.07–3.50)	0.028
Q4	2.52 (1.50–4.23)	<0.001	2.08 (1.22–3.57)	0.007	3.68 (1.91–7.09)	<0.001
*P* for trend	<0.001			<0.001		<0.001
**Premature death_2013**						
CTI (standardized)	1.74 (1.41–2.14)	<0.001	1.71 (1.35–2.16)	<0.001	2.61 (1.84–3.72)	<0.001
CTI						
Q1						
Q2	0.70 (0.27–1.83)	0.464	0.69 (0.26–1.82)	0.454	0.75 (0.28–2.00)	0.566
Q3	1.60 (0.73–3.53)	0.243	1.63 (0.73–3.64)	0.230	1.97 (0.85–4.55)	0.113
Q4	3.12 (1.53–6.36)	0.002	3.01 (1.41–6.40)	0.004	4.31 (1.75–10.59)	0.001
*P* for trend	<0.001			<0.001		<0.001
**All cause mortality_2020**						
CTI (standardized)	1.36 (1.19, 1.56)	<0.001	1.32 (1.14, 1.54)	<0.001	1.89 (1.50, 2.37)	<0.001
CTI						
Q1						
Q2	1.09 (0.67, 1.78)	0.721	0.96 (0.59, 1.57)	0.881	1.09 (0.66, 1.79)	0.739
Q3	1.42 (0.90, 2.25)	0.133	1.23 (0.77, 1.96)	0.379	1.55 (0.95, 2.54)	0.079
Q4	2.08 (1.36, 3.20)	<0.001	1.74 (1.11, 2.71)	0.015	2.74 (1.57, 4.76)	<0.001
*P* for trend	<0.001			0.005		<0.001
**Premature death_2020**						
CTI (standardized)	1.52 (1.26, 1.83)	<0.001	1.57 (1.28, 1.94) <0.001 2.15 (1.57, 2.95)	<0.001		
CTI						
Q1						
Q2	0.81 (0.39, 1.68)	0.571	0.83 (0.40, 1.73)	0.616	0.88 (0.42, 1.86)	0.742
Q3	1.38 (0.72, 2.62)	0.330	1.49 (0.77, 2.87)	0.232	1.70 (0.86, 3.37)	0.129
Q4	2.20 (1.22, 3.98)	0.009	2.38 (1.27, 4.46)	0.007	2.98 (1.38, 6.42)	0.005
*P* for trend	0.002			0.002		0.002

^1^HR, hazard ratio, CI, Confidence Interval; Model 1, no covariates were adjusted; Model 2, adjusted for age, gender, BMI, education, marital, hukou, smoking, drinking, HTN, DM, and CVD; Model 3, adjusted for age, gender, BMI, education, marital, hukou, smoking, drinking, HTN, DM, CVD, TG, LDL, HDL, UA, and GLU.

### 3.6 C-index analysis

**FIGURE 5:**
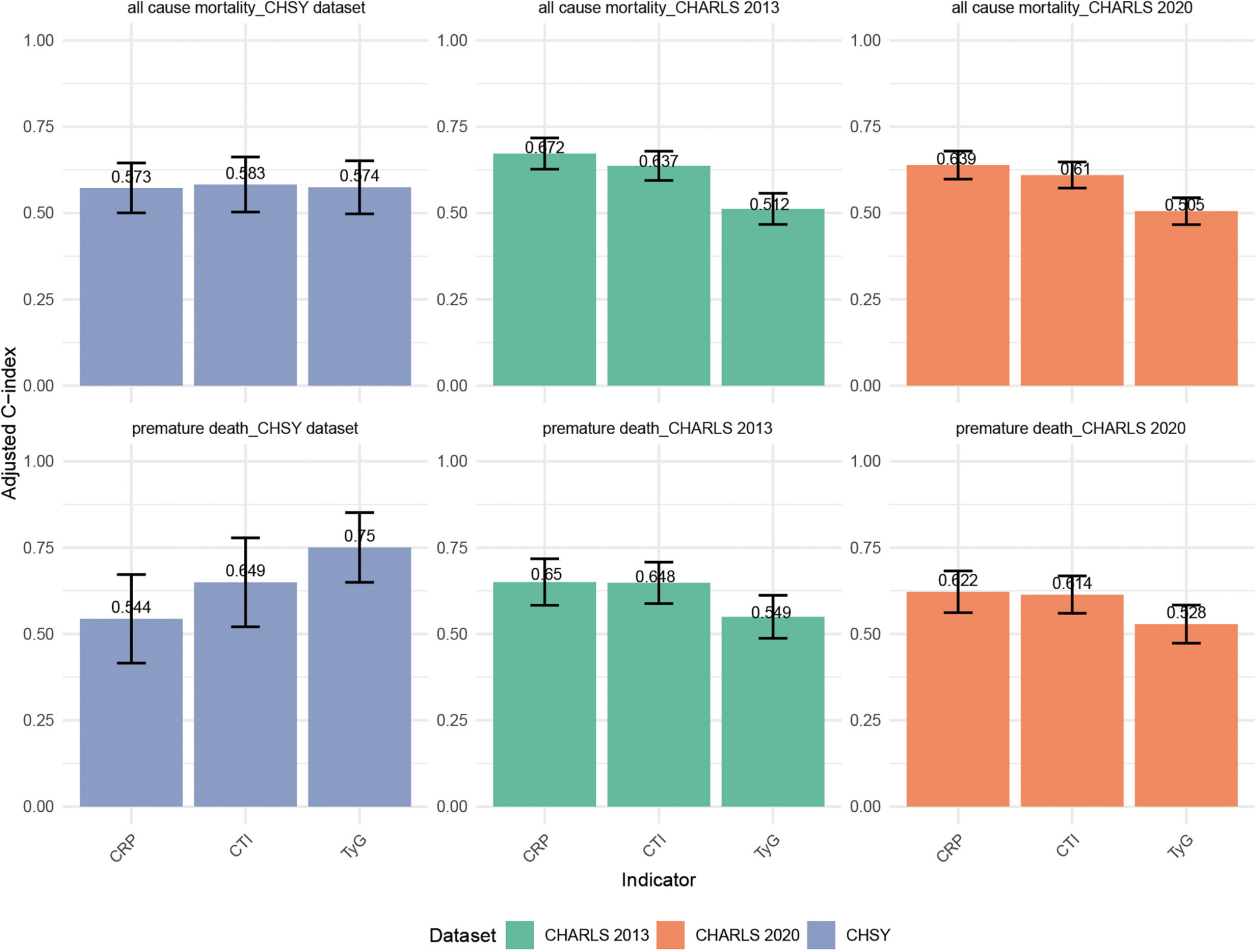
Comparison of C-indices for CTI, TyG, and CRP in predicting all-cause mortality and premature mortality across the CHARLS 2013, CHARLS 2020, and CHSY cohorts.

The discriminative ability of different indicators for all-cause mortality and premature mortality is shown in Figure 5. Overall, the discrimination of all three indicators was at a moderate level, with CTI performing slightly better than CRP and TyG in most scenarios, suggesting its potential advantage in risk prediction. In CHARLS 2013, the C-index of CTI for all-cause mortality was 0.637 (95% CI: 0.594–0.679), ranking intermediate among the three indicators—slightly lower than that of CRP (0.672) but significantly higher than that of TyG (0.512). For predicting premature mortality, CTI reached 0.648 (95% CI: 0.588–0.708), which was comparable to CRP (0.650), and both were markedly superior to TyG (0.549). In CHARLS 2020, the discrimination of CTI remained stable at approximately 0.61 for both outcomes (all-cause mortality: 0.610; premature mortality: 0.614), overall similar to CRP (0.639 and 0.622), and consistently higher than TyG (0.505 and 0.528). In the CHSY dataset, the differences in C-indices among the three indicators for all-cause mortality were small, though CTI was the highest (0.583, 95% CI: 0.503–0.662), slightly outperforming TyG (0.574) and CRP (0.573). Regarding premature mortality, although TyG had the highest C-index (0.750), CTI also reached 0.649 (95% CI: 0.521–0.778), outperforming CRP (0.544), indicating that it also possesses certain predictive utility in clinical populations.

## 4 Discussion

This study conducted a systematic evaluation of the relationship between CTI and the risks of all-cause and premature mortality, utilizing data from two extensive cohort studies: the CHARLS and CHSY. The findings indicated that an elevated CTI is significantly associated with increased risks of all-cause and premature mortality among middle-aged and older adults. These associations were consistently observed across different follow-up periods (2013 and 2020) and various population subgroups. The results suggest that CTI could serve as a critical biomarker for predicting mortality risk in individuals with metabolic-inflammatory dysregulation syndrome, underscoring its potential application in epidemiological research and clinical risk assessment.

Our findings extend the existing body of evidence regarding the TyG index. Although the TyG index has been widely recognized as a reliable marker of insulin resistance and a strong predictor of adverse health outcomes (12, 33), the CTI—a composite indicator integrating the TyG index with CRP, offers the advantage of capturing the synergistic effects of insulin resistance and systemic inflammation (34–36). Mechanistically, insulin resistance facilitates the accumulation of free fatty acids, which activates the NLRP3 inflammasome and triggers a systemic inflammatory cascade (37, 38). CRP, as an acute-phase protein, further exacerbates oxidative stress and endothelial dysfunction, ultimately contributing to organ damage and functional decline (39, 40). This study not only identified a significant linear dose-response relationship between CTI and mortality risk but also revealed a non-linear trend in the CHSY dataset. Restricted cubic spline analysis indicated that when CTI exceeded the threshold of 8.04–8.19, mortality risk exhibited an accelerated increase, suggesting the existence of a potential “critical point” in physiological compensatory mechanisms. This finding is consistent with the observations of Wang et al. regarding the “threshold effect” of CRP and provides a practical reference for mortality risk stratification (41).

Notably, this study conducted a joint analysis of two Chinese cohorts, CHARLS and CHSY. In addition to confirming the robust association between CTI and mortality risk in middle-aged and older adults, we also identified consistent associations between CTI and multiple adverse health conditions (e.g., poor self-rated health, chronic disease prevalence, and physical frailty) in the younger CHSY cohort. Although CHSY currently lacks long-term mortality data, its cross-sectional health indicators suggest that CTI may serve as an early warning biomarker for health risks, indicating its potential predictive value across different life stages. Next, we compared the differences in CTI thresholds between the CHARLS and CHSY cohorts. The analysis revealed that the CTI threshold associated with a significantly increased risk of mortality was slightly higher in CHARLS than in CHSY. This discrepancy may be attributed to differences in biological and demographic characteristics between the two cohorts: CHARLS represents a community-based middle-aged and elderly population with relatively good baseline health status, whereas CHSY consists of hospitalized middle-aged and elderly patients with a higher burden of chronic diseases. Consequently, additional risk may be observed at relatively lower CTI levels in the latter cohort. Furthermore, variations in laboratory testing conditions and data collection environments may also partially explain the differences in thresholds. Therefore, caution should be exercised when applying CTI thresholds across different cohorts or populations to avoid direct extrapolation. It is particularly noteworthy that in large-scale cohorts such as CHARLS, baseline differences between groups are often prone to achieving statistical significance due to the large sample size, yet this does not necessarily indicate clinical relevance. For instance, the lower LDL-C levels observed among deceased individuals in this study may not directly imply a reduced risk of mortality attributable to LDL-C itself. Instead, this finding is more likely to reflect underlying factors such as frailty, malnutrition, or the burden of chronic disorders.

This study also elucidated subgroup heterogeneity in the relationship between CTI and mortality risk. Educational attainment exhibits significant interactions with CTI at both follow-up intervals (2013 and 2020), with individuals possessing lower levels of education, particularly those with only an elementary school education or less, being more vulnerable to increased mortality risk due to metabolic-inflammatory dysregulation (42). This vulnerability may be closely linked to their lower health literacy, restricted access to healthcare, and unhealthy lifestyle behaviors (43, 44). Unlike Western cohorts such as the Health and Retirement Study (HRS), which predominantly focus on racial disparities, our findings underscore the pivotal role of educational attainment as a social determinant within the Chinese societal context. Furthermore, in the 2013 data, a significant interaction was observed between hypertension and diabetes status on the association of CTI with mortality risk. Surprisingly, the risk was higher in non-hypertensive and non-diabetic individuals. This phenomenon may reflect multiple underlying mechanisms: On one hand, some participants may have had undiagnosed hypertension or diabetes, where elevated CTI indicates early metabolic-inflammatory abnormalities. On the other hand, those with diagnosed chronic conditions may have received pharmacological treatment or lifestyle modifications, thereby attenuating short-term mortality risk (i.e., survivor bias). Additionally, reverse causality may also play a role—for instance, after diagnosis, patients with chronic diseases may adopt lifestyle changes or undergo disease management, leading to reduced short-term mortality and making the predictive effect of CTI on mortality more pronounced in non-chronic disease populations. In summary, these interpretations suggest that elevated CTI in populations without chronic diseases still warrants high attention, as it may indicate a potential high-risk state or unmanaged early metabolic-inflammatory burden. The attenuation of this interaction in 2020 may be related to overall improvements in health management.

The study presents several methodological innovations. By utilizing restricted cubic spline models, we elucidate the time-dependent non-linear relationship between CTI and mortality risk, thereby providing theoretical insights into the cumulative effects of metabolic-inflammatory burden. The integration of longitudinal data from the CHARLS with real-world data from the CHSY facilitates the development of a continuous health trajectory model, illustrating the impact of metabolic-inflammatory abnormalities on health outcomes across various life stages. Furthermore, the implementation of multiple imputation techniques, sensitivity analyses, and cross-validation across datasets effectively mitigates confounding bias, thereby enhancing the robustness of the findings and strengthening causal inference. It should be noted that in Model 3, we included metabolic indicators such as blood lipids (TG, LDL-C, HDL-C) and blood glucose (GLU), primarily considering their independent associations with mortality risk and their potential role as confounding factors influencing the relationship between CTI and mortality risk. However, these variables may also lie on the causal pathway, acting as potential mediators, and over-adjustment may lead to an underestimation of the effect of CTI. Therefore, we conducted supplementary sensitivity analyses by reconstructing the model without these metabolic indicators. The results showed that the direction and significance of the association between CTI and mortality risk remained consistent, indicating that the main conclusions are robust.

From a public health perspective, the CTI holds potential application value. The components of the CTI (CRP, triglycerides, and blood glucose) can be determined in most community and county-level healthcare institutions at a relatively low cost, making it feasible for large-scale population screening. However, certain barriers remain in low-resource settings, including limited availability of CRP testing equipment and reagents, issues related to laboratory standardization and unit harmonization, as well as insufficient training of healthcare personnel, which may affect the calculation of CTI and risk interpretation. Furthermore, the CTI thresholds identified in this study may only be applicable to specific Chinese populations. Caution should be exercised when extrapolating these values to other ethnic or lifestyle groups, and validation and local adaptation are recommended in different regions. Overall, as a composite metabolic-inflammatory indicator, the CTI shows potential advantages in public health screening and early risk identification. However, its practical implementation should be adapted according to local resource conditions and healthcare systems.

Several limitations warrant acknowledgement. Firstly, although comprehensive adjustments were made for numerous confounding factors, residual confounding may persist, particularly with regard to certain lifestyle variables such as dietary patterns, physical activity, and sleep quality. These should be addressed in future investigations. Secondly, while the CHSY cohort offers valuable insights into populations with early health disadvantages, the current absence of significant disease endpoint data somewhat limits its utility in mortality prediction. Thirdly, information on acute infection or inflammatory status was not systematically collected during the baseline survey; therefore, individuals with transiently elevated CRP levels due to acute inflammatory responses could not be fully excluded, which may introduce certain biases. Additionally, the calculation of CTI in this study relied on single baseline measurements and did not capture dynamic fluctuations in indicators such as CRP, blood glucose, and blood lipids. This may lead to an underestimation or overestimation of its predictive ability for long-term mortality risk. Future studies should incorporate clinical history, disease diagnoses, and repeated measurements to more comprehensively evaluate the relationship between dynamic changes in CTI and health outcomes. Furthermore, this study defined premature death based on the average life expectancy data in China at the 2011 baseline (72.7 years for males and 76.9 years for females). While this methodological approach is reasonable in design, it did not account for the slight increase in life expectancy during the follow-up period, which may have certain implications for the interpretation of the results. Therefore, although the findings of this study possess certain scientific significance and public health value, they are currently insufficient to be directly translated into clinical practice. Future multi-center, long-term prospective studies involving diverse populations are warranted, along with an evaluation of the cost-effectiveness and clinical feasibility of CTI, to determine its practical utility as a screening tool or risk prediction indicator.

## 5 Conclusion

This study, using data from two Chinese cohorts, found that a high composite metabolic-inflammatory index - CTI significantly raises the risk of all-cause mortality and early death, with consistent results across various groups and timeframes. The study highlights that individuals with lower education, males, and those without chronic disease diagnoses are more sensitive to CTI, enhancing its predictive value in high-risk groups. CTI is proposed as a valuable biomarker for assessing mortality risk. Future research should investigate the relationship between CTI changes and long-term health outcomes, as well as assess its applicability to broader populations. CTI can be integrated into public health strategies for improved risk assessment and personalized care.

## Data Availability

The raw data supporting the conclusions of this article will be made available by the authors, without undue reservation.
