# Supplementary material for: Association between C-reactive protein-triglyceride glucose index and all-cause mortality and premature death: a joint analysis based on case data from the Central Hospital of Shaoyang and CHARLS database
Source: Front Med (Lausanne). 2025 Oct 28;12:1656187. doi: 10.3389/fmed.2025.1656187 (PMC12602389; doi:10.3389/fmed.2025.1656187)
Supplement: Supplementary file 1 [file Table_1.docx]

Supplementary table 1. Description of the study variables.

| **SN** | **Predictors** | **Discription** | **Types** | **Values** |
| --- | --- | --- | --- | --- |
| **CHARLS dataset** | | | | |
| 1 | Gender | Sex of the patient | Categorical | 1: Male  2: Female |
| 2 | Age | Age is determined by calculating the difference between the year 2011 and the respondent's birth year. | Continuous | 45-96 |
| 3 | Drinking | If the respondent answers that they do not drink anything (including beer, wine, or liquor), they are defined as "non-drinking"; if they answer that they drink more than once a month or less than once a month, they are defined as "drinking." | Categorical | 0: Non-drinking  1: Drinking |
| 4 | Smoking | Smoking status is categorized into three distinct classifications on the basis of the respondent's response to the questionnaire. The respondents who indicated that they had never smoked (including cigarettes, pipes, or chewing tobacco) were classified as "Non-smokers." Those who reported having smoked in the past but have since ceased smoking are categorized as "Ex-smokers." Individuals who confirm current smoking habits are classified as "Smokers." | Categorical | 1: Non-smoker  2: Ex-smoker  3: Smoker |
| 5 | Diabetes (DM) | Diabetes is defined as a condition in which an individual is considered "diabetic" if their fasting glycated hemoglobin level is 6.5% or higher or if they have been diagnosed with diabetes or are taking medication for diabetes. Conversely, individuals with a fasting glycated hemoglobin level less than 6.5% who have not been diagnosed with diabetes and are not on diabetes medication are classified as "nondiabetic." | Categorical | 0: No diabetes  1: Diabetes |
| 6 | Hypertension  (HTN) | An individual is considered "nonhypertensive" if their systolic blood pressure is below 140 mmHg and their diastolic blood pressure is below 90 mmHg, provided that they have neither been diagnosed with hypertension nor received any treatment for it. Otherwise, the individual is classified as "hypertensive." | Categorical | 0: No hypertension  1: Hypertension |
| 7 | Coronary heart disease (CHD) | CVD is defined as the presence of a medical diagnosis of stroke or heart disease, which includes conditions such as myocardial infarction, coronary heart disease, angina pectoris, and congestive heart failure. Individuals with such a diagnosis are considered to have "cardiovascular disease," whereas those without such a diagnosis are considered "noncardiovascular disease." | Categorical | 0: No CHD  1: CHD |
| 8 | Body mass index (BMI) | BMI is a widely utilized metric for assessing the ratio of an individual's weight to height. It is calculated by dividing the individual's weight in kilograms by the square of their height in meters. | Continuous | 11.65- 134.47 |
| 9 | Education | Educational attainment is categorized into four levels on the basis of the response to "What is the highest level of education you have achieved? ": illiterate, primary school or below, middle school or above, and college/Uni+. | Categorical | 1: Illiterate  2: Primary  3: Second/high school  4: College/Uni+ |
| 10 | Marital | Marital status is categorized into four groups: married, divorced, widowed, and unmarried, based on the responses to the question "What is your current marital status?" | Categorical | 1: Married  2: Divorced  3: Widowed  4: Unmarried |
| 11 | Hukou | Hukou is categorized into two levels on the basis of the response to "What is the current hukou status": village and town. | Categorical | 1: Village  2: Town |
| 12 | TG | Triglycerides (mg/dl) | Continuous | 2.66-686.76 |
| 13 | LDL | Low density lipoprotein cholesterol (mg/dl) | Continuous | 5.41-286.08 |
| 14 | HDL | High density lipoprotein cholesterol (mg/dl) | Continuous | 3.09-127.19 |
| 15 | UA | Uric Acid (mg/dl) | Continuous | 0.26-10.68 |
| 16 | GLU | Glucose (mg/dl) | Continuous | 20.52-294.12 |
| **CHSY dataset** | | | | |
| 17 | TG^1^ | Triglycerides (mmol/L) | Continuous | 0.29-1.83 |
| 18 | LDL^2^ | Low density lipoprotein cholesterol (mmol/L) | Continuous | 0-3.36 |
| 19 | HDL^3^ | High density lipoprotein cholesterol (mmol/L) | Continuous | 0.91-2.19 |
| 20 | UA^4^ | Uric Acid (umol/L) | Continuous | 142-339 |
| 21 | GLU^5^ | Glucose (mmol/L) | Continuous | 3.60-6.10 |

^1^TG (mg/dl) = TG (mmol/L) * 88.57

^2^LDL (mg/dl) = LDL (mmol/L) * 38.66

^3^HDL (mg/dl) = HDL (mmol/L) * 38.66

^4^UA (mg/dl) = UA (umol/L) * 0.0168

^5^GLU (mg/dl) = GLU (mmol/L) * 18.02
