# Supplementary material for: Association between C-reactive protein-triglyceride glucose index and all-cause mortality and premature death: a joint analysis based on case data from the Central Hospital of Shaoyang and CHARLS database
Source: Front Med (Lausanne). 2025 Oct 28;12:1656187. doi: 10.3389/fmed.2025.1656187 (PMC12602389; doi:10.3389/fmed.2025.1656187)
Supplement: Supplementary file 2 [file Table_2.docx]

Supplementary table 2. Results of multicollinearity analysis in the CHARLS dataset.

| **Term** | **VIF** | **VIF_CI_low** | **VIF_CI_high** | **SE_factor** | **Tolerance** | **Tolerance_CI_low** | **Tolerance_CI_high** |
| --- | --- | --- | --- | --- | --- | --- | --- |
| Age | 1.461638 | 1.432335 | 1.497608 | 0.016428 | 0.684164 | 0.667732 | 0.698161 |
| Gender | 2.31811 | 2.25274 | 2.397249 | 0.036384 | 0.431386 | 0.417145 | 0.443904 |
| Education | 1.48827 | 1.461117 | 1.520884 | 0.015703 | 0.671921 | 0.657513 | 0.684408 |
| Marital | 1.118978 | 1.104891 | 1.138135 | 0.008258 | 0.893673 | 0.87863 | 0.905067 |
| Hukou | 1.201732 | 1.184148 | 1.22264 | 0.009968 | 0.832132 | 0.817902 | 0.844489 |
| Smoking | 1.788629 | 1.742862 | 1.849607 | 0.027904 | 0.559087 | 0.540655 | 0.573769 |
| Drinking | 1.359503 | 1.334017 | 1.389289 | 0.014243 | 0.735563 | 0.719792 | 0.749615 |
| HTN | 1.173899 | 1.15927 | 1.193559 | 0.008783 | 0.851862 | 0.83783 | 0.862612 |
| DM | 1.39272 | 1.344302 | 1.449736 | 0.026541 | 0.718019 | 0.689781 | 0.743881 |
| CVD | 1.063676 | 1.055539 | 1.076226 | 0.005362 | 0.940136 | 0.929173 | 0.947383 |
| BMI | 1.343824 | 1.321679 | 1.371579 | 0.013051 | 0.744145 | 0.729087 | 0.756614 |
| TG | 2.532469 | 2.462205 | 2.616766 | 0.039431 | 0.394872 | 0.382151 | 0.40614 |
| LDL | 1.082756 | 1.072212 | 1.097243 | 0.006461 | 0.923569 | 0.911375 | 0.932652 |
| HDL | 1.510708 | 1.484412 | 1.542441 | 0.015099 | 0.661941 | 0.648323 | 0.673667 |
| UA | 1.311231 | 1.290419 | 1.340483 | 0.012981 | 0.762642 | 0.746 | 0.774942 |
| GLU | 1.796966 | 1.732028 | 1.877573 | 0.037783 | 0.556493 | 0.532602 | 0.577358 |
| CTI | 3.242153 | 3.141155 | 3.360646 | 0.05608 | 0.308437 | 0.297562 | 0.318354 |
