# Supplementary material for: Association between C-reactive protein-triglyceride glucose index and all-cause mortality and premature death: a joint analysis based on case data from the Central Hospital of Shaoyang and CHARLS database
Source: Front Med (Lausanne). 2025 Oct 28;12:1656187. doi: 10.3389/fmed.2025.1656187 (PMC12602389; doi:10.3389/fmed.2025.1656187)
Supplement: Supplementary file 3 [file Table_3.docx]

Supplementary table 3. Results of normality tests in the CHARLS dataset.

| **Variable** | **Test** | **Statistic** | ***P -* value** |
| --- | --- | --- | --- |
| Age | Lilliefors (Kolmogorov-Smirnov) | 0.063336042 | 8.16E-113 |
| BMI | Lilliefors (Kolmogorov-Smirnov) | 0.037526987 | 4.19E-38 |
| TG | Lilliefors (Kolmogorov-Smirnov) | 0.154109742 | <0.001 |
| HDL | Lilliefors (Kolmogorov-Smirnov) | 0.053742575 | 2.22E-80 |
| LDL | Lilliefors (Kolmogorov-Smirnov) | 0.031651161 | 1.12E-26 |
| UA | Lilliefors (Kolmogorov-Smirnov) | 0.050729848 | 2.47E-71 |
| GLU | Lilliefors (Kolmogorov-Smirnov) | 0.199366373 | <0.001 |
| CTI | Lilliefors (Kolmogorov-Smirnov) | 0.042133459 | 1.64E-48 |
