# Supplementary material for: Association between C-reactive protein-triglyceride glucose index and all-cause mortality and premature death: a joint analysis based on case data from the Central Hospital of Shaoyang and CHARLS database
Source: Front Med (Lausanne). 2025 Oct 28;12:1656187. doi: 10.3389/fmed.2025.1656187 (PMC12602389; doi:10.3389/fmed.2025.1656187)
Supplement: Supplementary file 4 [file Table_4.docx]

Supplementary table 4. Patient demographics and baseline characteristics in the first wave of follow-up (2011-2013) in the CHARLS database.

| **Characteristic** |  | **All cause mortality_2013** | | | **Premature death_2013** | | |
| --- | --- | --- | --- | --- | --- | --- | --- |
|  | **Overall, N = 10,512^1^** | **No, N = 10,338^1^** | **Yes, N = 174^1^** | **SMD** | **No, N = 10,420^1^** | **Yes, N = 92^1^** | **SMD** |
| **Age** | 59.00 (52.00, 66.00) | 58.00 (52.00, 65.00) | 71.00 (62.00, 77.00) | 1.066^1^ | 59.00 (52.00, 66.00) | 62.00 (57.00, 67.50) | 0.298^1^ |
| **Gender** |  |  |  | 0.203^2^ |  |  | 0.216^2^ |
| Female | 5,576 (53.04%) | 5,501 (53.21%) | 75 (43.10%) |  | 5,537 (53.14%) | 39 (42.39%) |  |
| Male | 4,936 (46.96%) | 4,837 (46.79%) | 99 (56.90%) |  | 4,883 (46.86%) | 53 (57.61%) |  |
| **BMI** | 23.22 (20.89, 25.91) | 23.24 (20.91, 25.91) | 22.12 (19.03, 25.64) | 0.264^1^ | 23.22 (20.89, 25.91) | 23.29 (19.88, 25.93) | 0.095^1^ |
| **Education** |  |  |  | 0.412^3^ |  |  | 0.122^3^ |
| College/Uni+ | 162 (1.54%) | 161 (1.56%) | 1 (0.57%) |  | 161 (1.55%) | 1 (1.09%) |  |
| Illiterate | 3,070 (29.20%) | 2,990 (28.92%) | 80 (45.98%) |  | 3,042 (29.19%) | 28 (30.43%) |  |
| Primary | 4,261 (40.53%) | 4,196 (40.59%) | 65 (37.36%) |  | 4,220 (40.50%) | 41 (44.57%) |  |
| Second/high school | 3,019 (28.72%) | 2,991 (28.93%) | 28 (16.09%) |  | 2,997 (28.76%) | 22 (23.91%) |  |
| **Marital** |  |  |  | 0.416^3^ |  |  | 0.196^3^ |
| Divorced | 110 (1.05%) | 108 (1.04%) | 2 (1.15%) |  | 108 (1.04%) | 2 (2.17%) |  |
| Married | 9,244 (87.94%) | 9,118 (88.20%) | 126 (72.41%) |  | 9,169 (87.99%) | 75 (81.52%) |  |
| Unmarried | 88 (0.84%) | 86 (0.83%) | 2 (1.15%) |  | 86 (0.83%) | 2 (2.17%) |  |
| Widowed | 1,070 (10.18%) | 1,026 (9.92%) | 44 (25.29%) |  | 1,057 (10.14%) | 13 (14.13%) |  |
| **Hukou** |  |  |  | 0.005^2^ |  |  | 0.089^2^ |
| Town | 1,913 (18.20%) | 1,881 (18.20%) | 32 (18.39%) |  | 1,893 (18.17%) | 20 (21.74%) |  |
| Village | 8,599 (81.80%) | 8,457 (81.80%) | 142 (81.61%) |  | 8,527 (81.83%) | 72 (78.26%) |  |
| **Smoking** |  |  |  | 0.282^2^ |  |  | 0.376^2^ |
| Ex-smoker | 940 (8.94%) | 914 (8.84%) | 26 (14.94%) |  | 925 (8.88%) | 15 (16.30%) |  |
| Non-smoker | 6,439 (61.25%) | 6,355 (61.47%) | 84 (48.28%) |  | 6,399 (61.41%) | 40 (43.48%) |  |
| Smoker | 3,133 (29.80%) | 3,069 (29.69%) | 64 (36.78%) |  | 3,096 (29.71%) | 37 (40.22%) |  |
| **Drinking** |  |  |  | 0.065^2^ |  |  | 0.041^2^ |
| No | 7,058 (67.14%) | 6,936 (67.09%) | 122 (70.11%) |  | 6,998 (67.16%) | 60 (65.22%) |  |
| Yes | 3,454 (32.86%) | 3,402 (32.91%) | 52 (29.89%) |  | 3,422 (32.84%) | 32 (34.78%) |  |
| **HTN** |  |  |  | 0.370^2^ |  |  | 0.218^2^ |
| No | 5,927 (56.38%) | 5,860 (56.68%) | 67 (38.51%) |  | 5,885 (56.48%) | 42 (45.65%) |  |
| Yes | 4,585 (43.62%) | 4,478 (43.32%) | 107 (61.49%) |  | 4,535 (43.52%) | 50 (54.35%) |  |
| **DM** |  |  |  | 0.201^2^ |  |  | 0.322^2^ |
| No | 9,606 (91.38%) | 9,458 (91.49%) | 148 (85.06%) |  | 9,532 (91.48%) | 74 (80.43%) |  |
| Yes | 906 (8.62%) | 880 (8.51%) | 26 (14.94%) |  | 888 (8.52%) | 18 (19.57%) |  |
| **CVD** |  |  |  | 0.224^2^ |  |  | 0.270^2^ |
| No | 8,991 (85.53%) | 8,857 (85.67%) | 134 (77.01%) |  | 8,922 (85.62%) | 69 (75.00%) |  |
| Yes | 1,521 (14.47%) | 1,481 (14.33%) | 40 (22.99%) |  | 1,498 (14.38%) | 23 (25.00%) |  |
| **TG** | 106.20 (75.23, 156.65) | 106.20 (75.23, 156.65) | 106.20 (77.00, 153.11) | 0.029^1^ | 106.20 (75.23, 156.65) | 115.49 (78.77, 158.86) | 0.077^1^ |
| **LDL** | 114.05 (92.78, 136.86) | 114.05 (93.17, 137.24) | 104.77 (83.12, 126.81) | 0.200^1^ | 114.05 (92.98, 136.86) | 104.58 (80.80, 128.74) | 0.251^1^ |
| **HDL** | 49.10 (40.21, 59.92) | 49.10 (40.21, 59.92) | 48.13 (38.66, 58.76) | 0.069^1^ | 49.10 (40.21, 59.92) | 45.62 (35.57, 56.44) | 0.254^1^ |
| **UA** | 4.31 (3.57, 5.18) | 4.31 (3.57, 5.18) | 4.42 (3.64, 5.48) | 0.142^1^ | 4.31 (3.57, 5.18) | 4.42 (3.62, 5.45) | 0.120^1^ |
| **GLU** | 102.42 (94.41, 113.94) | 102.42 (94.32, 113.76) | 105.75 (95.22, 122.76) | 0.230^1^ | 102.42 (94.32, 113.76) | 109.53 (98.01, 125.19) | 0.381^1^ |
| **CTI** | 8.69 (8.17, 9.30) | 8.69 (8.17, 9.28) | 9.15 (8.54, 9.69) | 0.444^1^ | 8.69 (8.17, 9.29) | 9.21 (8.65, 9.85) | 0.559^1^ |

^1^Median (IQR); n (%)

^2^Wilcoxon rank sum test

^3^Pearson's Chi-squared test

^4^Fisher's exact test
