# Supplementary material for: Association between C-reactive protein-triglyceride glucose index and all-cause mortality and premature death: a joint analysis based on case data from the Central Hospital of Shaoyang and CHARLS database
Source: Front Med (Lausanne). 2025 Oct 28;12:1656187. doi: 10.3389/fmed.2025.1656187 (PMC12602389; doi:10.3389/fmed.2025.1656187)
Supplement: Supplementary file 5 [file Table_5.docx]

Supplementary table 5. Patient demographics and baseline characteristics in the second wave of follow-up (2011-2020) in the CHARLS database.

| **Characteristic** |  | **All cause mortality_2020** | | | **Premature death_2020** | | |
| --- | --- | --- | --- | --- | --- | --- | --- |
|  | **Overall, N = 10,512^1^** | **No, N = 10,283^1^** | **Yes, N = 229^1^** | **SMD** | **No, N = 10,397^1^** | **Yes, N = 115^1^** | **SMD** |
| **Age** | 59.00 (52.00, 66.00) | 58.00 (52.00, 65.00) | 70.00 (62.00, 76.00) | 1.046^1^ | 59.00 (52.00, 66.00) | 62.00 (57.00, 67.00) | 0.258^1^ |
| **Gender** |  |  |  | 0.229^2^ |  |  | 0.249^2^ |
| Female | 5,576 (53.04%) | 5,480 (53.29%) | 96 (41.92%) |  | 5,529 (53.18%) | 47 (40.87%) |  |
| Male | 4,936 (46.96%) | 4,803 (46.71%) | 133 (58.08%) |  | 4,868 (46.82%) | 68 (59.13%) |  |
| **BMI** | 23.22 (20.89, 25.91) | 23.25 (20.92, 25.92) | 21.97 (19.37, 25.51) | 0.279^1^ | 23.23 (20.90, 25.91) | 22.62 (19.80, 25.64) | 0.191^1^ |
| **Education** |  |  |  | 0.430^3^ |  |  | 0.140^3^ |
| College/Uni+ | 162 (1.54%) | 161 (1.57%) | 1 (0.44%) |  | 161 (1.55%) | 1 (0.87%) |  |
| Illiterate | 3,070 (29.20%) | 2,965 (28.83%) | 105 (45.85%) |  | 3,034 (29.18%) | 36 (31.30%) |  |
| Primary | 4,261 (40.53%) | 4,173 (40.58%) | 88 (38.43%) |  | 4,210 (40.49%) | 51 (44.35%) |  |
| Second/high school | 3,019 (28.72%) | 2,984 (29.02%) | 35 (15.28%) |  | 2,992 (28.78%) | 27 (23.48%) |  |
| **Marital** |  |  |  | 0.469^3^ |  |  | <0.306^3^ |
| Divorced | 110 (1.05%) | 106 (1.03%) | 4 (1.75%) |  | 107 (1.03%) | 3 (2.61%) |  |
| Married | 9,244 (87.94%) | 9,084 (88.34%) | 160 (69.87%) |  | 9,154 (88.04%) | 90 (78.26%) |  |
| Unmarried | 88 (0.84%) | 83 (0.81%) | 5 (2.18%) |  | 83 (0.80%) | 5 (4.35%) |  |
| Widowed | 1,070 (10.18%) | 1,010 (9.82%) | 60 (26.20%) |  | 1,053 (10.13%) | 17 (14.78%) |  |
| **Hukou** |  |  |  | 0.004^2^ |  |  | 0.002^2^ |
| Town | 1,913 (18.20%) | 1,871 (18.20%) | 42 (18.34%) |  | 1,892 (18.20%) | 21 (18.26%) |  |
| Village | 8,599 (81.80%) | 8,412 (81.80%) | 187 (81.66%) |  | 8,505 (81.80%) | 94 (81.74%) |  |
| **Smoking** |  |  |  | 0.310^2^ |  |  | 0.385^2^ |
| Ex-smoker | 940 (8.94%) | 904 (8.79%) | 36 (15.72%) |  | 924 (8.89%) | 16 (13.91%) |  |
| Non-smoker | 6,439 (61.25%) | 6,331 (61.57%) | 108 (47.16%) |  | 6,390 (61.46%) | 49 (42.61%) |  |
| Smoker | 3,133 (29.80%) | 3,048 (29.64%) | 85 (37.12%) |  | 3,083 (29.65%) | 50 (43.48%) |  |
| **Drinking** |  |  |  | 0.089^2^ |  |  | 0.004^2^ |
| No | 7,058 (67.14%) | 6,895 (67.05%) | 163 (71.18%) |  | 6,981 (67.14%) | 77 (66.96%) |  |
| Yes | 3,454 (32.86%) | 3,388 (32.95%) | 66 (28.82%) |  | 3,416 (32.86%) | 38 (33.04%) |  |
| **HTN** |  |  |  | 0.318^2^ |  |  | 0.138^2^ |
| No | 5,927 (56.38%) | 5,833 (56.72%) | 94 (41.05%) |  | 5,870 (56.46%) | 57 (49.57%) |  |
| Yes | 4,585 (43.62%) | 4,450 (43.28%) | 135 (58.95%) |  | 4,527 (43.54%) | 58 (50.43%) |  |
| **DM** |  |  |  | 0.174^2^ |  |  | 0.243^2^ |
| No | 9,606 (91.38%) | 9,409 (91.50%) | 197 (86.03%) |  | 9,510 (91.47%) | 96 (83.48%) |  |
| Yes | 906 (8.62%) | 874 (8.50%) | 32 (13.97%) |  | 887 (8.53%) | 19 (16.52%) |  |
| **CVD** |  |  |  | 0.229^2^ |  |  | 0.255^2^ |
| No | 8,991 (85.53%) | 8,815 (85.72%) | 176 (76.86%) |  | 8,904 (85.64%) | 87 (75.65%) |  |
| Yes | 1,521 (14.47%) | 1,468 (14.28%) | 53 (23.14%) |  | 1,493 (14.36%) | 28 (24.35%) |  |
| **TG** | 106.20 (75.23, 156.65) | 106.20 (75.23, 157.53) | 104.43 (75.23, 151.34) | 0.054^1^ | 106.20 (75.23, 156.65) | 108.86 (77.88, 153.11) | 0.048^1^ |
| **LDL** | 114.05 (92.78, 136.86) | 114.43 (93.17, 137.24) | 103.61 (83.12, 126.42) | 0.218^1^ | 114.05 (93.17, 136.86) | 105.93 (81.57, 129.90) | 0.225^1^ |
| **HDL** | 49.10 (40.21, 59.92) | 49.10 (40.21, 59.92) | 48.71 (39.82, 59.92) | 0.011^1^ | 49.10 (40.21, 59.92) | 47.94 (37.50, 58.76) | 0.107^1^ |
| **UA** | 4.31 (3.57, 5.18) | 4.31 (3.57, 5.17) | 4.58 (3.65, 5.57) | 0.202^1^ | 4.31 (3.57, 5.18) | 4.45 (3.60, 5.44) | 0.128^1^ |
| **GLU** | 102.42 (94.41, 113.94) | 102.42 (94.32, 113.76) | 105.84 (95.22, 123.84) | 0.232^1^ | 102.42 (94.32, 113.76) | 109.26 (97.38, 123.84) | 0.323^1^ |
| **CTI** | 8.69 (8.17, 9.30) | 8.69 (8.17, 9.28) | 9.11 (8.43, 9.65) | 0.356^1^ | 8.69 (8.17, 9.29) | 9.12 (8.54, 9.70) | 0.436^1^ |

^1^Median (IQR); n (%)

^2^Wilcoxon rank sum test

^3^Pearson's Chi-squared test

^4^Fisher's exact test
