# Supplementary material for: Association between C-reactive protein-triglyceride glucose index and all-cause mortality and premature death: a joint analysis based on case data from the Central Hospital of Shaoyang and CHARLS database
Source: Front Med (Lausanne). 2025 Oct 28;12:1656187. doi: 10.3389/fmed.2025.1656187 (PMC12602389; doi:10.3389/fmed.2025.1656187)
Supplement: Supplementary file 6 [file Table_6.docx]

Supplementary table 6. Patient demographics and baseline characteristics in the CHSY database.

| **Variables** | **CTI** | | | | | | | **SMD** |
| --- | --- | --- | --- | --- | --- | --- | --- | --- |
|  | **Q1  N = 461** | | **Q2  N = 460** | | **Q3  N = 460** | **Q4  N = 461** | |  |
| **Age, Median (Q1, Q3)** | 65.00 (55.00, 74.00) | | 65.50 (56.00, 74.50) | | 65.00 (56.00, 74.00) | 66.00 (55.00, 73.00) | | 0.028^1^ |
| **Gender, n (%)** |  | |  | |  |  | | 0.023^2^ |
| Male | 257 (55.75%) | | 257 (55.87%) | | 248 (53.91%) | 253 (54.88%) | |  |
| Female | 204 (44.25%) | | 203 (44.13%) | | 212 (46.09%) | 208 (45.12%) | |  |
| **BMI, Median (Q1, Q3)** | 20.93 (17.16, 25.36) | | 21.47 (17.25, 25.51) | | 21.41 (17.27, 26.05) | 21.75 (17.55, 25.61) | | 0.051^1^ |
| **Hukou, n (%)** |  | |  | |  |  | | 0.091^2^ |
| Town | 220 (47.72%) | | 244 (53.04%) | | 245 (53.26%) | 212 (45.99%) | |  |
| Village | 241 (52.28%) | | 216 (46.96%) | | 215 (46.74%) | 249 (54.01%) | |  |
| **Smoking, n (%)** |  | |  | |  |  | | 0.097^2^ |
| Ex-smoker | 145 (31.45%) | | 163 (35.43%) | | 146 (31.74%) | 162 (35.14%) | |  |
| Non-smoker | 163 (35.36%) | | 158 (34.35%) | | 169 (36.74%) | 138 (29.93%) | |  |
| Smoker | 153 (33.19%) | | 139 (30.22%) | | 145 (31.52%) | 161 (34.92%) | |  |
| **Drinking, n (%)** |  | |  | |  |  | | 0.043^2^ |
| No | 221 (47.94%) | | 232 (50.43%) | | 237 (51.52%) | 222 (48.16%) | |  |
| Yes | 240 (52.06%) | | 228 (49.57%) | | 223 (48.48%) | 239 (51.84%) | |  |
| **HTN, n (%)** |  | |  | |  |  | | 0.028^2^ |
| No | 227 (49.24%) | | 239 (51.96%) | | 232 (50.43%) | 231 (50.11%) | |  |
| Yes | 234 (50.76%) | | 221 (48.04%) | | 228 (49.57%) | 230 (49.89%) | |  |
| **DM, n (%)** |  | |  | |  |  | | 0.030^2^ |
| No | 220 (47.72%) | | 232 (50.43%) | | 226 (49.13%) | 222 (48.16%) | |  |
| Yes | 241 (52.28%) | 228 (49.57%) | | 234 (50.87%) | | | 239 (51.84%) |  |
| **CVD, n (%)** |  |  | |  | | |  | 0.069^2^ |
| No | 218 (47.29%) | 247 (53.70%) | | 221 (48.04%) | | | 217 (47.07%) |  |
| Yes | 243 (52.71%) | 213 (46.30%) | | 239 (51.96%) | | | 244 (52.93%) |  |
| **TG, Median (Q1, Q3)** | 0.93 (0.73, 1.21) | 1.21 (0.85, 1.63) | | 1.28 (0.94, 1.94) | | | 1.78 (1.27, 2.77) | 0.704^1^ |
| **LDL, Median (Q1, Q3)** | 2.43 (1.97, 2.94) | 2.57 (2.01, 3.08) | | 2.60 (2.06, 3.11) | | | 2.59 (2.03, 3.14) | 0.110^1^ |
| **HDL, Median (Q1, Q3)** | 1.17 (0.99, 1.35) | 1.08 (0.90, 1.28) | | 1.04 (0.89, 1.26) | | | 0.99 (0.80, 1.19) | 0.300^1^ |
| **UA, Median (Q1, Q3)** | 294.00 (240.80, 354.70) | 306.50 (236.20, 389.90) | | 304.25 (240.85, 376.30) | | | 320.50 (234.90, 410.00) | 0.109^1^ |
| **GLU, Median (Q1, Q3)** | 4.81 (4.35, 5.52) | 5.23 (4.52, 6.25) | | 5.49 (4.80, 6.80) | | | 7.23 (5.63, 9.89) | 0.678^1^ |

^1^Kruskal-Wallis rank sum test

^2^Pearson's Chi-squared test
