# Supplementary material for: Association between C-reactive protein-triglyceride glucose index and all-cause mortality and premature death: a joint analysis based on case data from the Central Hospital of Shaoyang and CHARLS database
Source: Front Med (Lausanne). 2025 Oct 28;12:1656187. doi: 10.3389/fmed.2025.1656187 (PMC12602389; doi:10.3389/fmed.2025.1656187)
Supplement: Supplementary file 7 [file Table_7.docx]

Supplementary table 7. Hazard ratios (HRs) of all-cause and premature mortality associated with CTI, presented as per-unit and per-standard-deviation (per-SD) increases, across three Cox proportional hazards models in the CHARLS 2013, CHARLS 2020, and CHSY datasets.

| **Dataset** | **Model** | **Variable** | **HR (95% CI)** |
| --- | --- | --- | --- |
| All-cause mortality in CHARLS 2013 dataset |  |  |  |
|  | Model 1 | CTI (per-unit) | 1.65 (1.40-1.94) |
|  | Model 1 | CTI (per-SD) | 1.52 (1.33-1.74) |
|  | Model 2 | CTI (per-unit) | 1.67 (1.39-2.00) |
|  | Model 2 | CTI (per-SD) | 1.53 (1.31-1.79) |
|  | Model 3 | CTI (per-unit) | 2.50 (1.92-3.25) |
|  | Model 3 | CTI (per-SD) | 2.15 (1.73-2.68) |
| Premature mortality in CHARLS 2013 dataset |  |  |  |
|  | Model 1 | CTI (per-unit) | 1.91 (1.53–2.38) |
|  | Model 1 | CTI (per-SD) | 1.72 (1.43–2.06) |
|  | Model 2 | CTI (per-unit) | 1.89 (1.48–2.41) |
|  | Model 2 | CTI (per-SD) | 1.70 (1.39–2.09) |
|  | Model 3 | CTI (per-unit) | 2.90 (2.03–4.15) |
|  | Model 3 | CTI (per-SD) | 2.44 (1.81–3.29) |
| All-cause mortality in CHARLS 2020 dataset |  |  |  |
|  | Model 1 | CTI (per-unit) | 1.50 (1.30–1.73) |
|  | Model 1 | CTI (per-SD) | 1.40 (1.25–1.58) |
|  | Model 2 | CTI (per-unit) | 1.52 (1.29–1.79) |
|  | Model 2 | CTI (per-SD) | 1.42 (1.24–1.63) |
|  | Model 3 | CTI (per-unit) | 2.10 (1.66–2.65) |
|  | Model 3 | CTI (per-SD) | 1.86 (1.53–2.26) |
| Premature mortality in CHARLS 2020 dataset |  |  |  |
|  | Model 1 | CTI (per-unit) | 1.67 (1.37–2.04) |
|  | Model 1 | CTI (per-SD) | 1.54 (1.30–1.82) |
|  | Model 2 | CTI (per-unit) | 1.74 (1.40–2.18) |
|  | Model 2 | CTI (per-SD) | 1.59 (1.32–1.92) |
|  | Model 3 | CTI (per-unit) | 2.50 (1.80–3.46) |
|  | Model 3 | CTI (per-SD) | 2.15 (1.64–2.82) |
| All-cause mortality in CHSY dataset |  |  |  |
|  | Model 1 | CTI (per-unit) | 1.70 (1.33–2.15) |
|  | Model 1 | CTI (per-SD) | 1.64 (1.31–2.05) |
|  | Model 2 | CTI (per-unit) | 1.86 (1.44–2.40) |
|  | Model 2 | CTI (per-SD) | 1.79 (1.40–2.27) |
|  | Model 3 | CTI (per-unit) | 1.92 (1.48–2.48) |
|  | Model 3 | CTI (per-SD) | 1.84 (1.44–2.34) |
| Premature mortality in CHSY dataset |  |  |  |
|  | Model 1 | CTI (per-unit) | 2.49 (1.74–3.55) |
|  | Model 1 | CTI (per-SD) | 2.34 (1.68–3.27) |
|  | Model 2 | CTI (per-unit) | 2.56 (1.77–3.68) |
|  | Model 2 | CTI (per-SD) | 2.40 (1.71–3.38) |
|  | Model 3 | CTI (per-unit) | 2.52 (1.75–3.62) |
|  | Model 3 | CTI (per-SD) | 2.37 (1.69–3.33) |
