# Supplementary material for: Association between C-reactive protein-triglyceride glucose index and all-cause mortality and premature death: a joint analysis based on case data from the Central Hospital of Shaoyang and CHARLS database
Source: Front Med (Lausanne). 2025 Oct 28;12:1656187. doi: 10.3389/fmed.2025.1656187 (PMC12602389; doi:10.3389/fmed.2025.1656187)
Supplement: Supplementary file 8 [file Table_8.docx]

Supplementary table 8. AIC of different nodes in RCS analysis.

| Final Incident | AIC for different nodes | | | | | The final node selected |
| --- | --- | --- | --- | --- | --- | --- |
|  | 3 | 4 | 5 | 6 | 7 |  |
| All cause mortality_CHARLS 2013 | 3189.3007 | 3187.5801 | 3189.5039 | 3191.5294 | 3192.4704 | 4 |
| Premature death_ CHARLS 2013 | 1674.9780 | 1675.1344 | 1677.0100 | 1678.4847 | 1679.8713 | 3 |
| All cause mortality_ CHARLS 2020 | 4210.9692 | 4210.0406 | 4211.9189 | 4213.5616 | 4215.1987 | 4 |
| Premature death_ CHARLS 2020 | 2107.2965 | 2108.5353 | 2109.3639 | 2111.2583 | 2112.7758 | 3 |
