# Supplementary material for: Association between C-reactive protein-triglyceride glucose index and all-cause mortality and premature death: a joint analysis based on case data from the Central Hospital of Shaoyang and CHARLS database
Source: Front Med (Lausanne). 2025 Oct 28;12:1656187. doi: 10.3389/fmed.2025.1656187 (PMC12602389; doi:10.3389/fmed.2025.1656187)
Supplement: Supplementary file 9 [file Table_9.docx]

Supplementary table 9. Relationship between CTI and survival after excluding chronically Ill patients.

| **Characteristic** | **Model 1** | | **Model 2** | | **Model 3** | |
| --- | --- | --- | --- | --- | --- | --- |
|  | **HR (95% CI)^1^** | ***p*** | **HR (95% CI)^1^** | ***p*** | **HR (95% CI)^1^** | ***p*** |
| **All cause mortality_2013** |  |  |  |  |  |  |
| CTI (standardized) | 1.71 (1.35–2.18) | <0.001 | 1.98 (1.52–2.57) | <0.001 | 2.72 (1.91–3.87) | <0.001 |
| **Premature death_2013** |  |  |  |  |  |  |
| CTI (standardized) | 2.07 (1.51–2.84) | <0.001 | 2.35 (1.69–3.27) | <0.001 | 3.11 (1.96–4.94) | <0.001 |
| **All cause mortality_2020** |  |  |  |  |  |  |
| CTI (standardized) | 1.41 (1.15–1.74) | <0.001 | 1.52 (1.21–1.90) | <0.001 | 2.21 (1.63–3.00) | <0.001 |
| **Premature death_2020** |  |  |  |  |  |  |
| CTI (standardized) | 1.59 (1.21–2.09) | <0.001 | 1.76 (1.33–2.33) | <0.001 | 2.34 (1.58–3.47) | <0.001 |

^1^HR = Hazard Ratio, CI = Confidence Interval; Model 1: No covariates were adjusted; Model 2: Adjusted for Age, Gender, BMI, Education, Marital, Hukou, Smoking, and Drinking; Model 3: Adjusted for Age, Gender, BMI, Education, Marital, Hukou, Smoking, Drinking, TG, LDL, HDL, UA, and GLU.
