# Supplementary material for: Association between C-reactive protein-triglyceride glucose index and all-cause mortality and premature death: a joint analysis based on case data from the Central Hospital of Shaoyang and CHARLS database
Source: Front Med (Lausanne). 2025 Oct 28;12:1656187. doi: 10.3389/fmed.2025.1656187 (PMC12602389; doi:10.3389/fmed.2025.1656187)
Supplement: Supplementary file 10 [file Table_10.docx]

Supplementary table 10. Sensitivity analysis excluding specific variables.

| **Characteristic** | **Model 1** | | **Model 2** | | **Model 3** | |  |
| --- | --- | --- | --- | --- | --- | --- | --- |
|  | **HR (95% CI)** | ***p*** | **HR (95% CI)** | ***p*** | **HR (95% CI)** | ***p*** |  |
| **All cause mortality_2013 in CHARLS dataset** |  |  |  |  |  |  |  |
| CTI (standardized) | 1.52 (1.33–1.74) | <0.001 | 1.53 (1.31–1.79) | <0.001 | 1.56 (1.33–1.82) | <0.001 |  |
| **Premature death_2013 in CHARLS dataset** |  |  |  |  |  |  |  |
| CTI (standardized) | 1.72 (1.43–2.06) | <0.001 | 1.70 (1.39–2.09) | <0.001 | 1.72 (1.40–2.13) | <0.001 |  |
| **All cause mortality_2020 in CHARLS dataset** |  |  |  |  |  |  |  |
| CTI (standardized) | 1.40 (1.25–1.58) | <0.001 | 1.42 (1.24–1.63) | <0.001 | 1.42 (1.23–1.63) | <0.001 |  |
| **Premature death_2020 in CHARLS dataset** |  |  |  |  |  |  |  |
| CTI (standardized) | 1.54 (1.30–1.82) | <0.001 | 1.59 (1.32–1.92) | <0.001 | 1.60 (1.32–1.93) | <0.001 |  |
| **All cause mortality in CHSY dataset** |  |  |  |  |  |  |  |
| CTI (standardized) | | 1.64 (1.31–2.05) | <0.001 | 1.79 (1.40–2.27) | <0.001 | 1.78 (1.40–2.27) | <0.001 |
| **Premature death in CHSY dataset** |  |  |  |  |  |  |  |
| CTI (standardized) | 2.34 (1.68–3.27) | <0.001 | 2.40 (1.71–3.38) | <0.001 | 2.37 (1.68–3.35) | <0.001 |  |

HR = Hazard Ratio, CI = Confidence Interval; Model 1: No covariates were adjusted, Model 2 in CHARLS dataset: adjusted for Age, Gender, BMI, Education, Marital, Hukou, Smoking, Drinking, HTN, DM, and CVD, Model 3 in CHARLS dataset: adjusted for Age, Gender, BMI, Education, Marital, Hukou, Smoking, Drinking, HTN, DM, CVD, and UA. Model 2 in CHSY dataset: adjusted for Age, Gender, BMI, Hukou, Smoking, Drinking, HTN, DM, and CVD, Model 3 in CHSY dataset: adjusted for Age, Gender, BMI, Hukou, Smoking, Drinking, HTN, DM, CVD, and UA.
