# Supplementary figures and images for: Association between C-reactive protein-triglyceride glucose index and all-cause mortality and premature death: a joint analysis based on case data from the Central Hospital of Shaoyang and CHARLS database
Source: Front Med (Lausanne). 2025 Oct 28;12:1656187. doi: 10.3389/fmed.2025.1656187 (PMC12602389; doi:10.3389/fmed.2025.1656187)

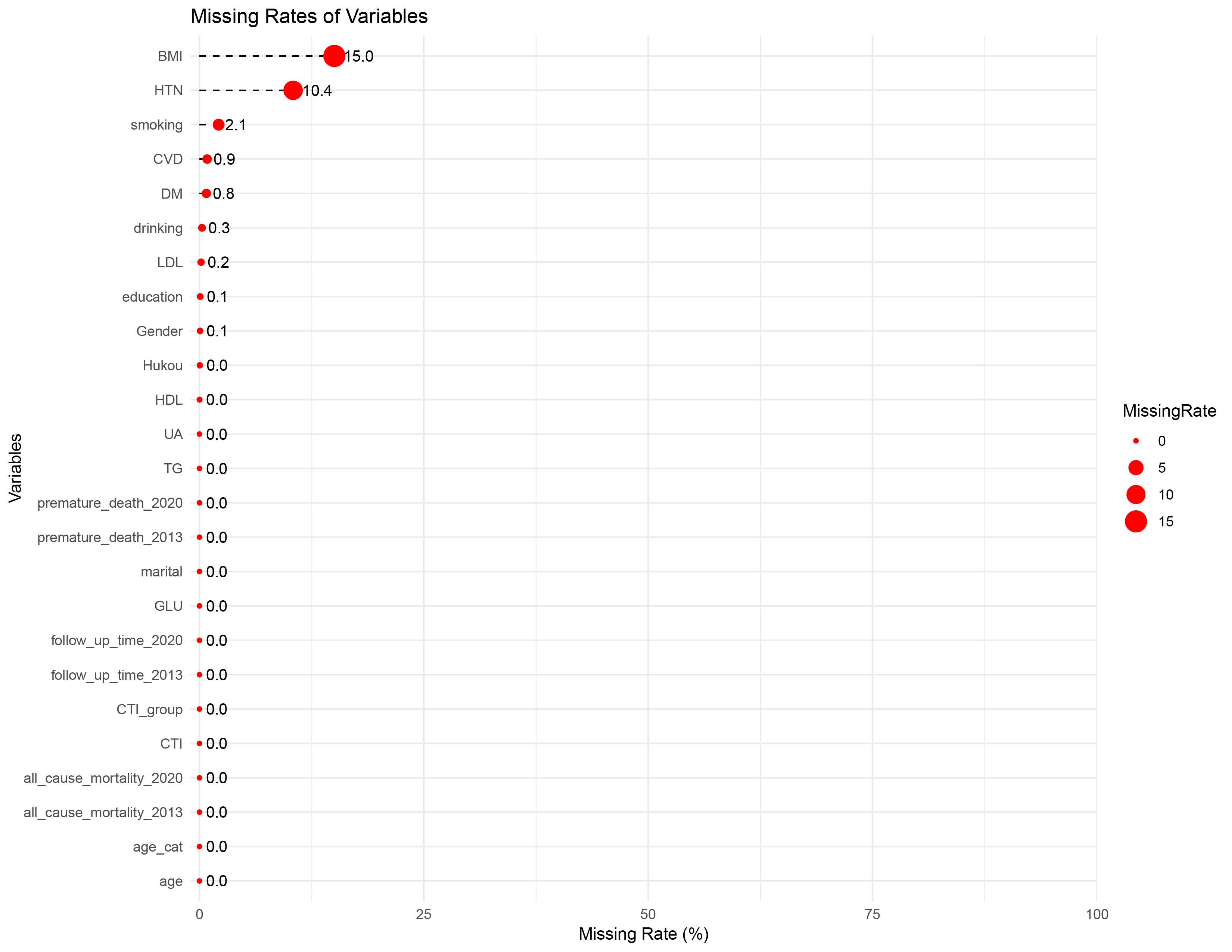

Supplement: Supplementary file 11 [file Image_1.tif]

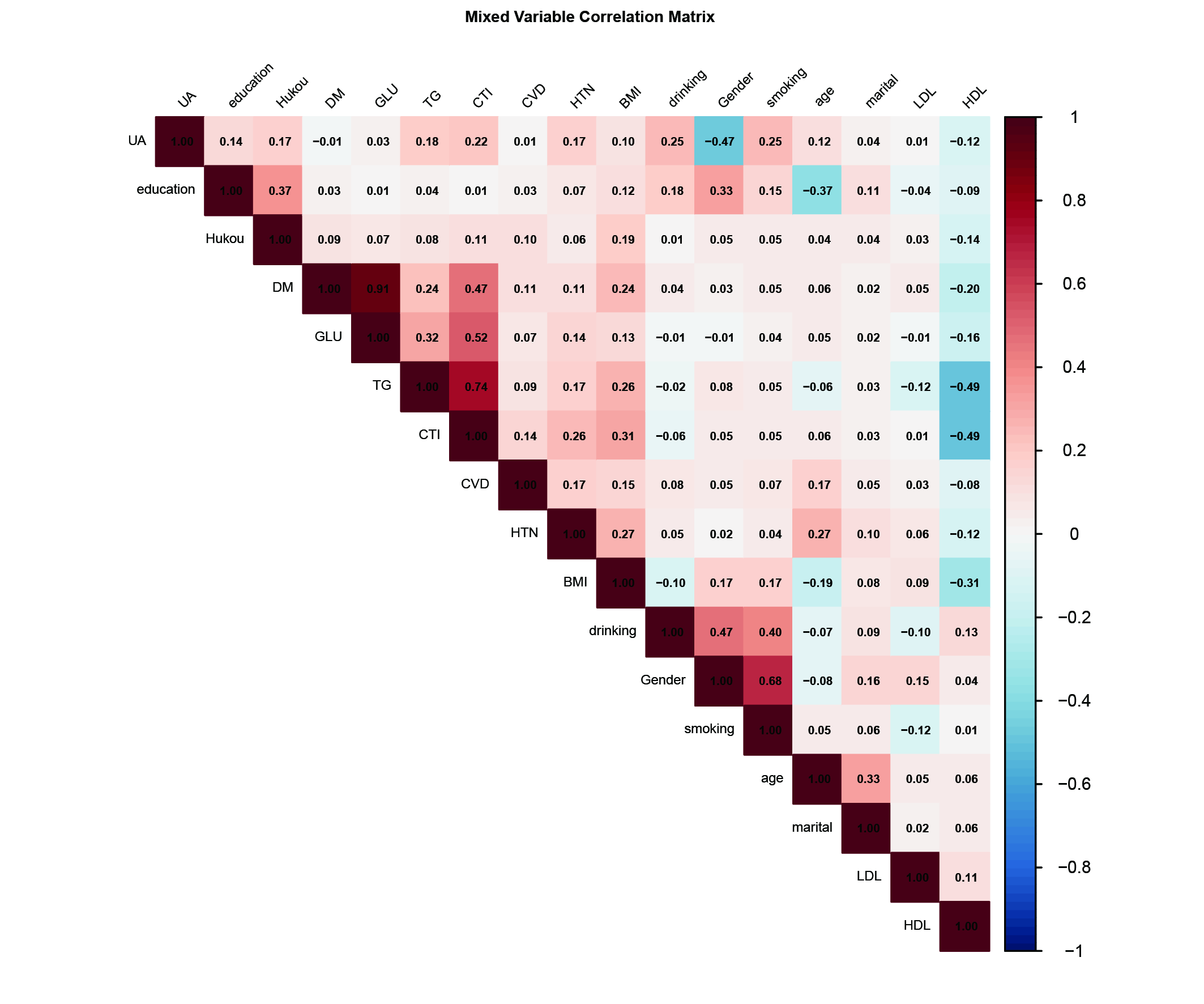

Supplement: Supplementary file 12 [file Image_2.tif]

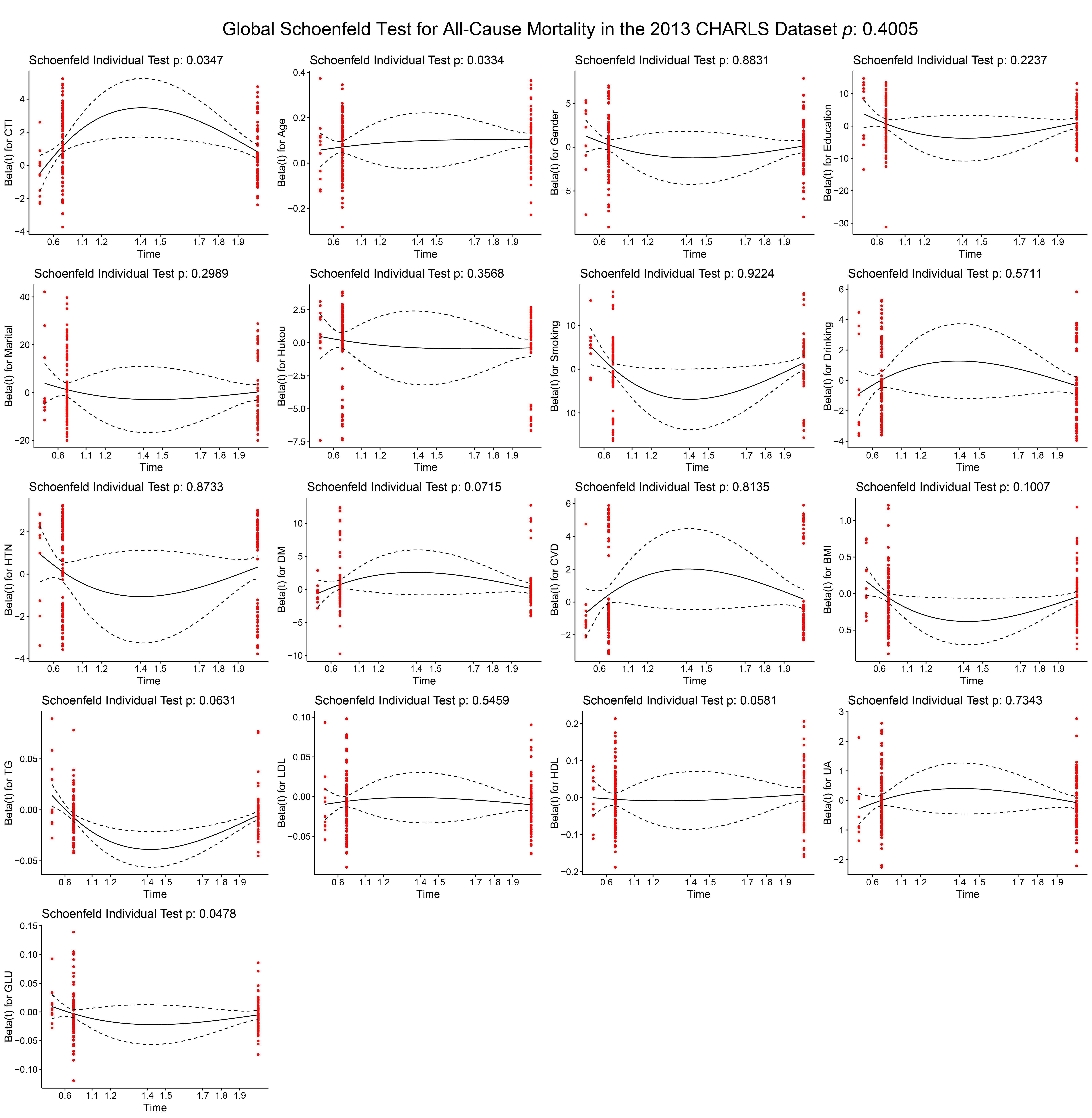

Supplement: Supplementary file 13 [file Image_3.tif]

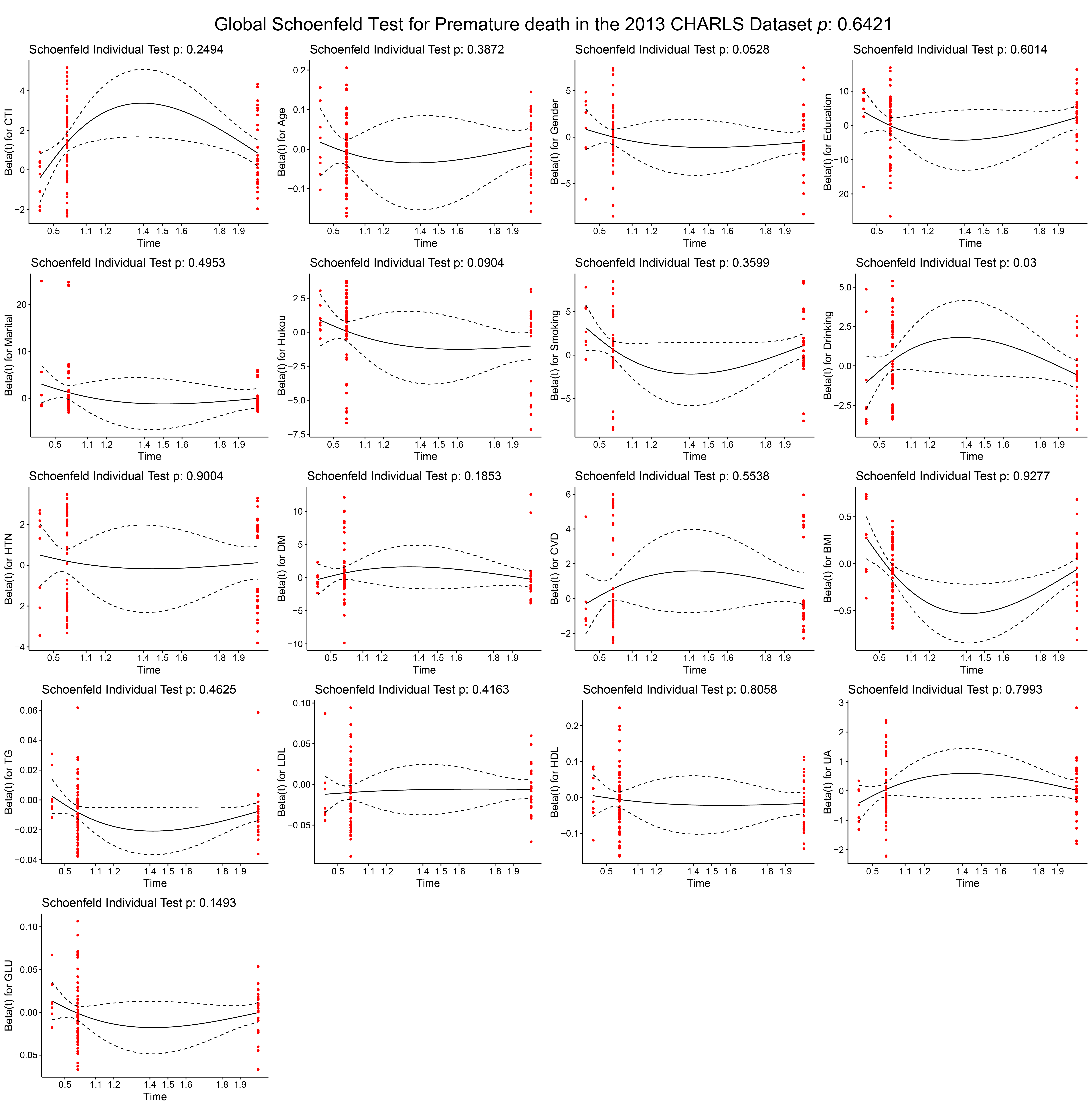

Supplement: Supplementary file 14 [file Image_4.tif]

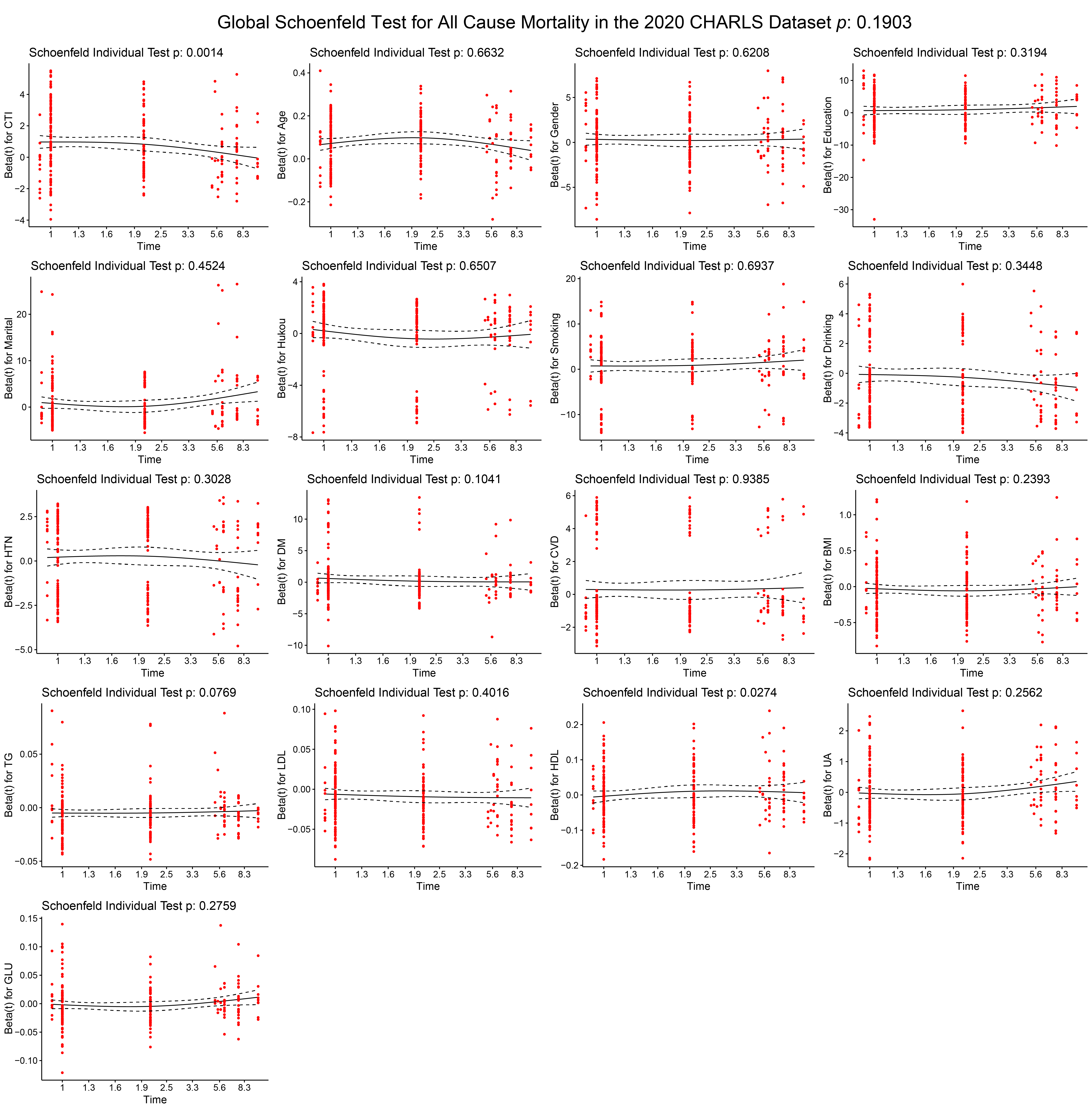

Supplement: Supplementary file 15 [file Image_5.tif]

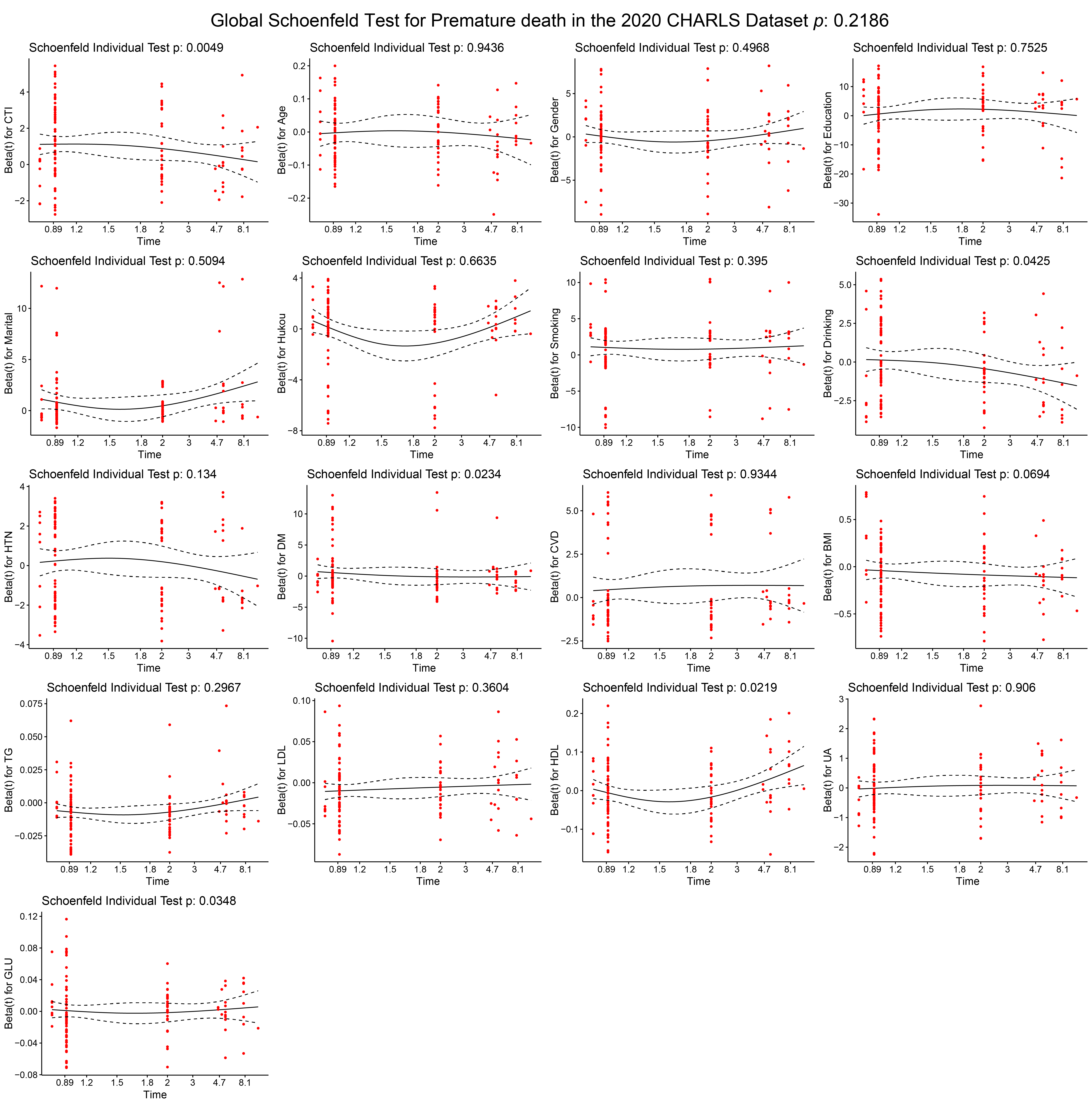

Supplement: Supplementary file 16 [file Image_6.tif]

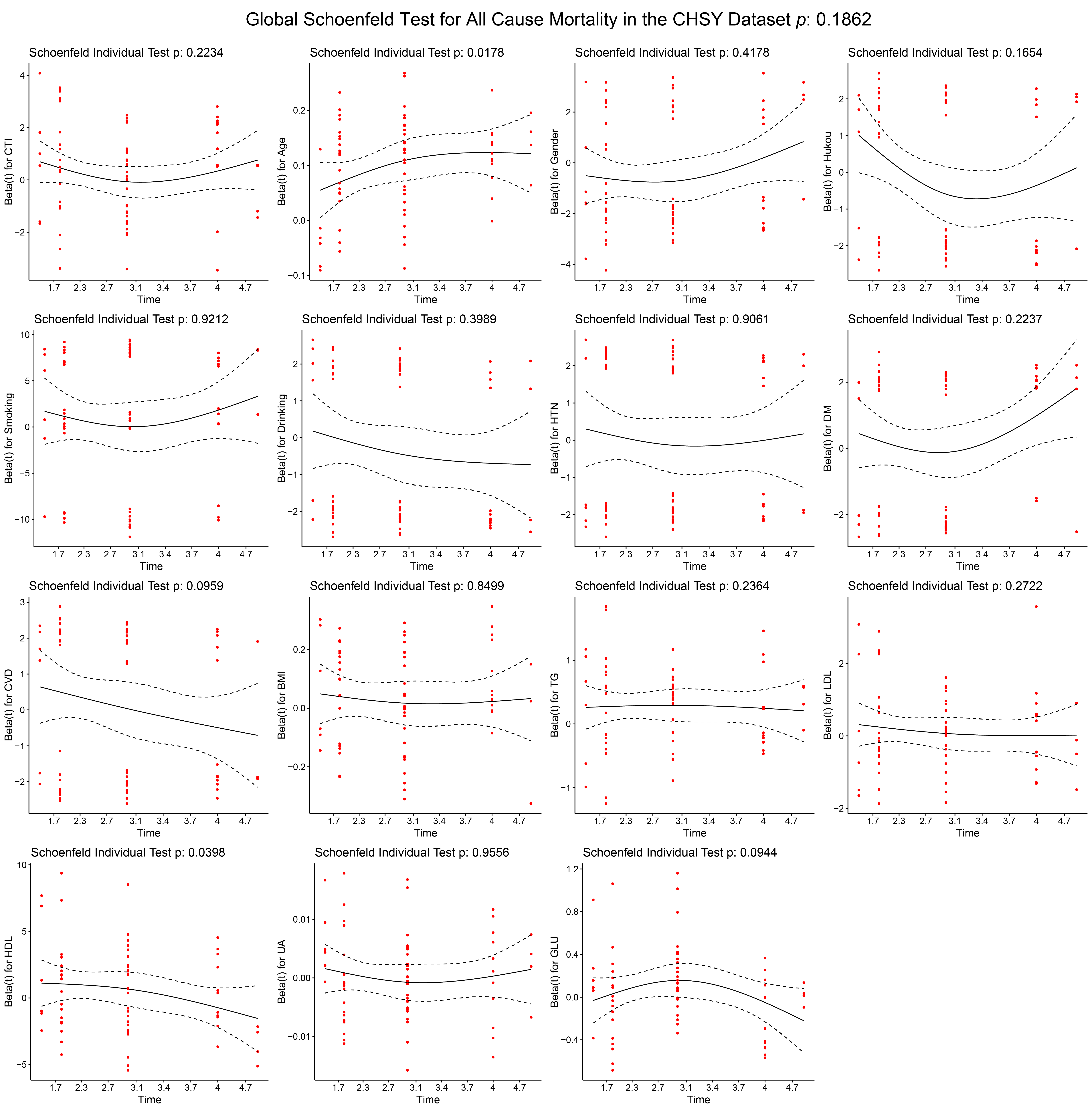

Supplement: Supplementary file 17 [file Image_7.tif]

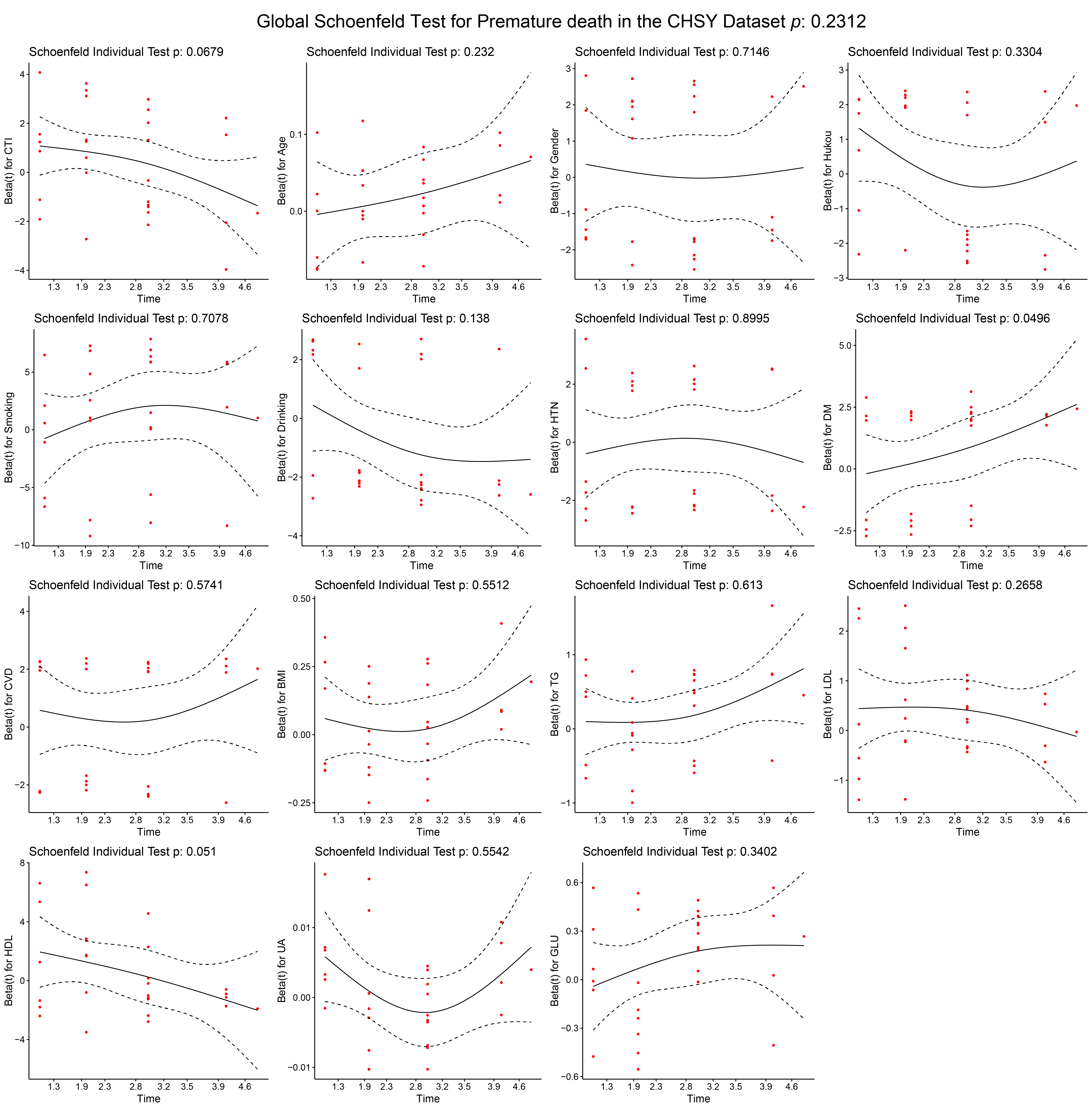

Supplement: Supplementary file 18 [file Image_8.tif]

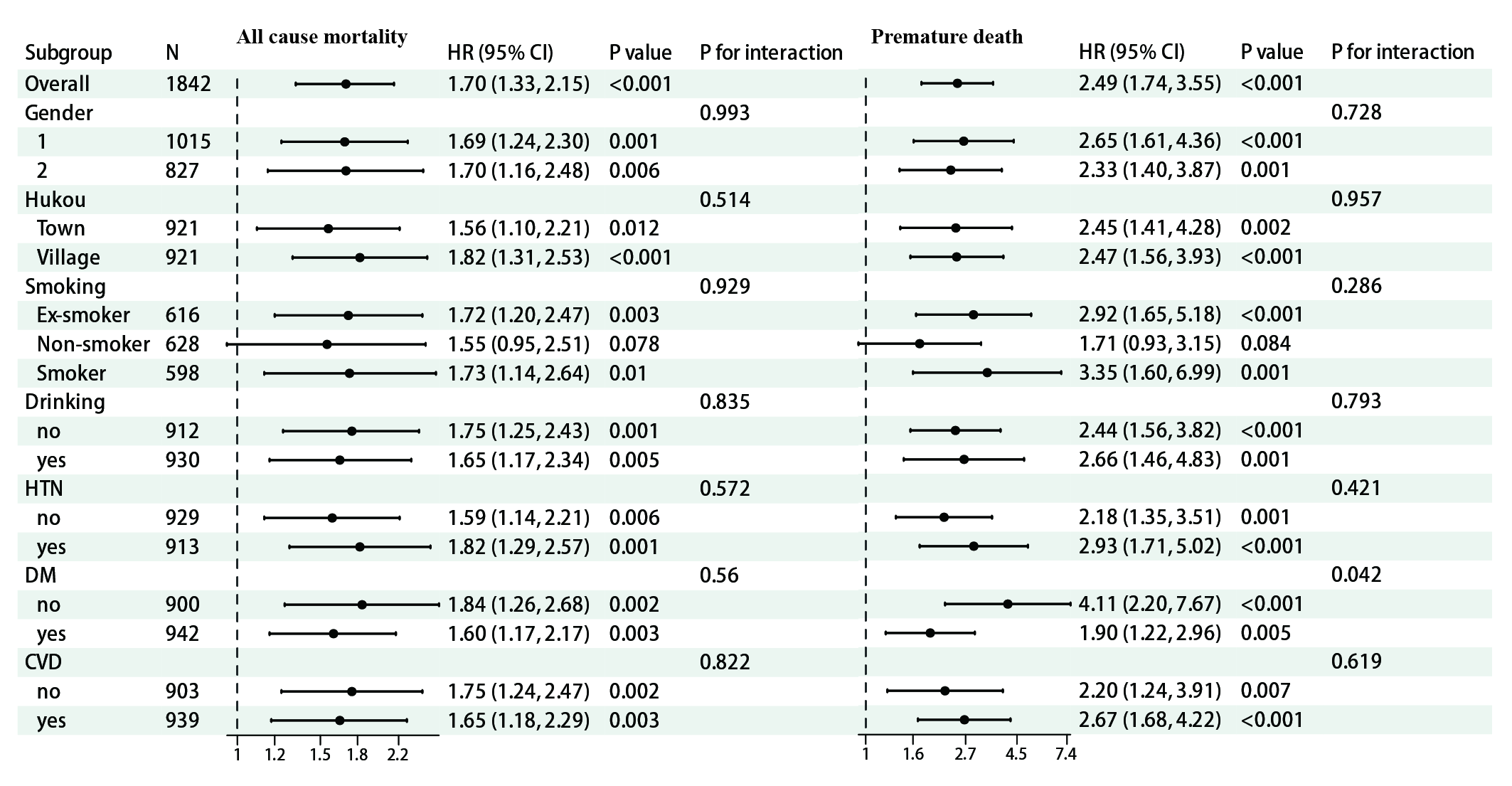

Supplement: Supplementary file 19 [file Image_9.tif]
